# Clinical and Research MRI Techniques for Assessing Spinal Cord Integrity in Degenerative Cervical Myelopathy—A Scoping Review

**DOI:** 10.3390/biomedicines10102621

**Published:** 2022-10-18

**Authors:** Brandon He, Kyle Sheldrick, Abhirup Das, Ashish Diwan

**Affiliations:** 1 Spine Labs, St. George & Sutherland Clinical School, UNSW Faculty of Medicine, Kogarah, NSW 2217, Australia; 2 Faculty of Medicine, University of New South Wales, Kensington, NSW 2052, Australia; 3 Spine Service, Department of Orthopaedic Surgery, St. George Hospital Campus, Kogarah, NSW 2217, Australia

**Keywords:** cervical spine, degenerative cervical myelopathy (DCM), cervical spondylotic myelopathy (CSM), spinal cord compression, quantitative MRI

## Abstract

Background: Degenerative cervical myelopathy (DCM) manifests as the primary cause of spinal cord dysfunction and is non-traumatic, chronic and progressive in nature. Decompressive surgery is typically utilised to halt further disability and neurological dysfunction. The limitations of current diagnostic options surrounding assessment and prognostic potential render DCM still largely a clinical diagnosis. Aims: To outline the limitations of current diagnostic techniques, present evidence behind novel quantitative MRI (qMRI) techniques for assessing spinal cord integrity in DCM and suggest future directions. Method: Articles published up to November 2021 were retrieved from Medline, EMBASE and EBM using key search terms: spinal cord, spine, neck, MRI, magnetic resonance imaging, qMRI, T1, T2, T2*, R2*, DTI, diffusion tensor imaging, MT, magnetisation transfer, SWI, susceptibility weighted imaging, BOLD, blood oxygen level dependent, fMRI, functional magnetic resonance imaging, functional MRI, MRS, magnetic resonance spectroscopy. Results: A total of 2057 articles were retrieved with 68 articles included for analysis. The search yielded 2 articles on Quantitative T1 mapping which suggested higher T1 values in spinal cord of moderate-severe DCM; 43 articles on DTI which indicated a strong correlation of fractional anisotropy and modified Japanese Orthopaedic Association scores; 15 articles on fMRI (BOLD) which demonstrated positive correlation of functional connectivity and volume of activation of various connections in the brain with post-surgical recovery; 6 articles on MRS which suggested that Choline/N-acetylaspartate (Cho/NAA) ratio presents the best correlation with DCM severity; and 4 articles on MT which revealed a preliminary negative correlation of magnetisation transfer ratio with DCM severity. Notably, most studies were of low sample size with short timeframes within 6 months. Conclusions: Further longitudinal studies with higher sample sizes and longer time horizons are necessary to determine the full prognostic capacity of qMRI in DCM.

## 1. Introduction

### 1.1. Epidemiology

Degenerative cervical myelopathy (DCM), earlier referred to as cervical spondylotic myelopathy (CSM), is the most common cause of spinal cord dysfunction, affecting an estimated 5% of adults over 40 years of age [1]. It is a significant cause of disability and carries substantial economic burden to the affected individuals, including their families and their community [2].

A comprehensive review of the literature demonstrated that such degenerative conditions of the spine are estimated to encompass 59% of non-traumatic spinal cord injury in Japan [3], 54% in the United States [4], 18–26% in Australia [5,6,7] and 16–39% in Europe [8,9,10,11,12,13,14]. The incidence was also purported to be 76, 26 and 6 per million in North America, Europe and Australia, respectively [7]. Notably, these data exclude many patients with less severe symptoms. Very few studies have been conducted on DCM prevalence. In Victoria, Australia, the prevalence of all non-traumatic spinal cord injury was estimated to be 367.2 per million in 2010 [15]. On the basis of these figures, studies have estimated the incidence and prevalence of DCM in the United States to be 41 and 605 per million, respectively [16].

However, a constraining factor of these estimates are the poor quality of the data of which they are derived from, and it is likely that the figures severely underestimate the burden of disease. As one of the most common causes of non-traumatic spinal-cord injury it is reasonable to infer that DCM represents a formidable issue in the aging population [17].

### 1.2. Natural History

**Figure 1:**
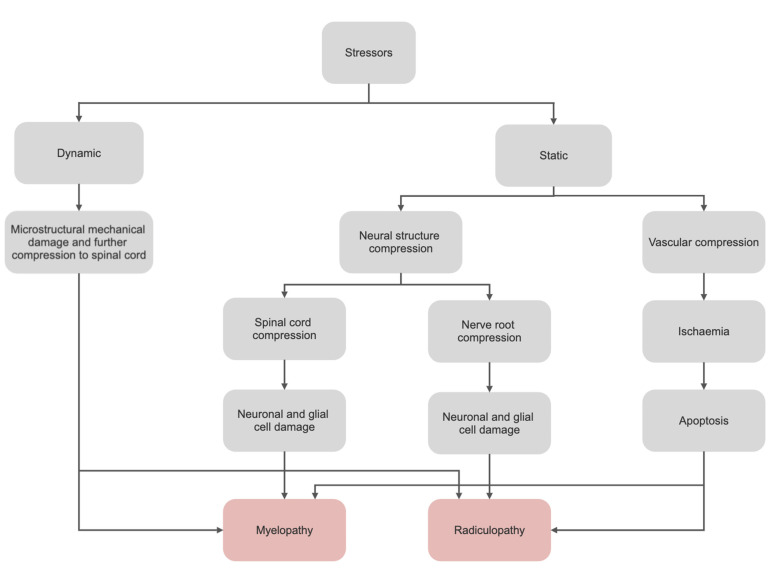
The pathogenesis of DCM. A combination of dynamic and static stressors is purported to contribute to the development of DCM. Neural structure compression includes spinal canal compression, spine deformity, disc herniation, osteophyte formation, ossification of the posterior longitudinal ligaments (OPLL) and ossification of the ligamentum flavum (OFL). Dynamic stressors include biomechanical changes, invagination of the ligamentum flavum and microstructural mechanical spinal cord damage from cervical instability. Abbreviations: DCM, degenerative cervical myelopathy.

AO Spine RECODE-DCM has recently listed the diagnosis and pathogenesis, as some of the top priorities in DCM research [18]. Degenerative cervical myelopathy is a degenerative condition and as such, it is non-traumatic, chronic and progressive in nature, with surgery traditionally utilised to halt further disability and neurological dysfunction [19]. The pathogenesis of DCM is purported to involve a myriad of static and dynamic factors (Figure 1). Static factors include spinal canal compression, spine deformity, disc herniation, osteophyte formation, ossification of the posterior longitudinal ligaments (OPLL) and ossification of the ligamentum flavum (OFL). Owing to its mobility, the vertebral column of the neck also suffers from dynamic stressors that include biomechanical changes, invagination of the ligamentum flavum and microstructural mechanical spinal cord damage from cervical instability. Such stressors, at a chronic magnitude, induce direct neuronal and glial cell damage as well as a secondary ischaemic cascade of neuronal excitotoxicity and apoptosis which contribute to the development of DCM [17].

Notably, very little is currently known with regards to the exact molecular mechanisms underlying the process of this condition. A 1963 retrospective study of the natural history of DCM found that a majority of patients had poor prognosis, 87% of which progressing to moderate or severe disability at the last follow up. Several historical and present day studies have indicated that the disease course of DCM is highly variable [20,21,22,23,24,25,26,27,28,29,30,31,32,33]. In particular, a 1956 study of 120 DCM patients and found 5% patients had a rapid onset of symptoms followed by long periods of quiescence, 20% had a slow, progressive deleteriousness of neurofunction, and 75% had a stepwise decline of neurofunction [21]. A further 2017 systematic review demonstrated that 20–67% of patients had experienced neurological deterioration after 3–6 years of follow-up [34]. It is not yet clear what manifests these differences in DCM pathogenesis between patients. Further research into DCM at a molecular level could result in promising diagnostic methods, enable detection at early stages and thus render timely intervention and treatment.

The current data associated with the natural history of DCM is largely derived from low-quality retrospective studies [19,35]. The limited existing prospective studies are markedly underpowered and have low level evidence with inconsistent results and risks of bias [36]. Accordingly, there persists a necessity for a large-scale prospective study focusing on natural history of DCM, specifically with the inclusion of novel multiparametric quantitative MRI that will be discussed further on.

### 1.3. Current Diagnostic Options and Limitations

#### 1.3.1. Clinical

**Figure 2:**
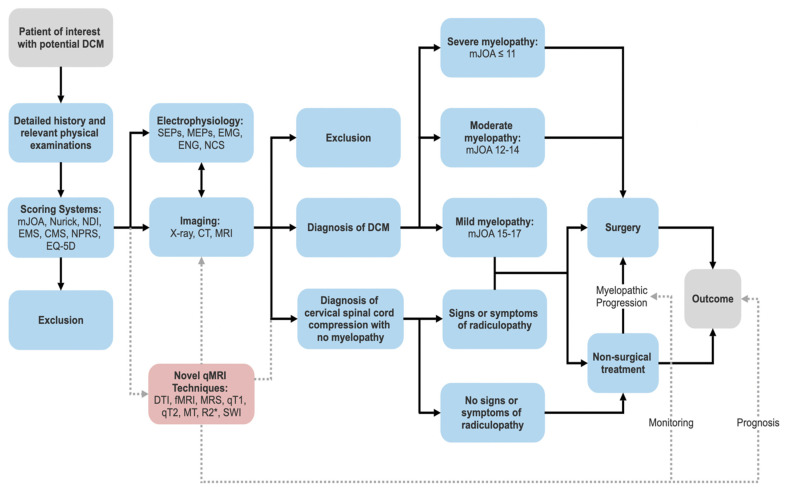
Where novel qMRI would fit into the current diagnostic work-up and treatment of degenerative cervical myelopathy. The dashed lines represent pathways currently under investigation. The current diagnostic work-up starts with a detailed history, physical examination, and application of scoring systems. Electrophysiology and imaging can rule out differentials and provide additional information to generate a diagnosis of DCM or cervical spinal cord compression without myelopathy. Surgery is recommended for moderate to severe myelopathy according to current guidelines. Patients with spinal cord compression and evidence of radiculopathy may be offered surgical or non-surgical treatment. Patients with spinal cord compression and no evidence of radiculopathy should undergo clinical monitoring. Surgery may be offered to patients utilising non-operative treatment upon worsening of condition. There is potential for qMRI to play a role in monitoring this progression and provide prognostic value to the outcome of DCM. Abbreviations: CMS, cervical myelopathy score; CT, computed tomography; DCM, Degenerative cervical myelopathy; DTI, Diffusion tensor imaging; EMG, electromyography; EMS, European myelopathy score; ENG, electroneurography; fMRI, functional MRI; MEPs, motor evoked potentials; mJOA, modified Japanese orthopaedic association score; MRI, magnetic resonance imaging; MRS, magnetic resonance spectroscopy; MT, magnetization transfer; NCS, nerve conduction studies; NDI, neck disability index; NPRS, numeric pain rating scale; qMRI, quantitative magnetic resonance imaging; qT1, quantitative T1; qT2, quantitative T2; SEPs, somatosensory evoked potentials; SWI, susceptibility weighted imaging.

Diagnosis of DCM typically necessitates a congruity between clinical (Table 1.) and investigatory findings (Figure 2). A thorough history and physical examination should first ensue when DCM is suspected.

Limitations: Although a useful element of diagnosis, physical tests are not always consistent in their ability to quantify the severity of DCM which is an important element in the consideration of surgical intervention [44]. Thus, correlations in further investigatory measures are required to arrive at the correct diagnosis.

#### 1.3.2. Scoring Systems

Clinicians utilise scoring systems to categories the functional impairment of various conditions. Whilst different classifications may arise, there typically exists one standardised system for publications and treatments. This is not true for DCM; whereby different systems are utilised based upon preference. A 2016 systematic-review revealed that reported outcomes varied widely between studies of DCM [45]. Table 2 details the current most common classification systems, their benefits and limitations. The mJOA scale followed by the Nurick Grading system are the current most widely adopted measure for DCM patients [46].

However, the limitations covered in Table 2, are particularly problematic in mild DCM whereby strong floor and ceiling effects^[e]^ in these scales inhibit ascertainment of more subtle neurological changes that provide information for decision-making in surgery18. As such, there exists the need to develop both a standardised scoring system and more sensitive and objective outcome instruments to enable more effective clinical assessment and efficient synthesis of research.

#### 1.3.3. Conventional MRI

Conventional MRI is the primary modality utilised for imaging in DCM as it enables high-resolution depiction of neural structures, bone and ligaments that are difficult to visualise in other scans [57]. Conventional MRI (such as T1-weighted and T2-weighted imaging) can characterise the degree and nature of degeneration (i.e., OPLL, spondylosis, disc herniation, hypertrophy of ligamentum flavum), identify spinal-cord compression, highlight changes in spinal-canal diameter, and detect changes in signal intensity [58,59,60]. MRI can also assist in ruling-out resembling differentials or other causes of myelopathy such as a tumour syringomyelia or demyelinating plaques [2,38,42]. CT myelography should be utilised in situations of MRI contraindication [61].

Identifying spinal-cord compression plays a pivotal role in treatment selection and outcome prediction and thus should be the foremost investigation. It is typically described based on the number of compression sites [30], appearance [32,62,63,64,65] or ratio between the anteroposterior diameter and the transverse diameter (CR = Compression Ratio) [66,67]. A maximum spinal-cord compression (MSCC) index has also been developed by Fehlings et al. as a measurement of spinal-cord compression [68]. The primary object of these methods is to determine severity of spinal-cord compression.

Measurements of the anterior-posterior diameter at the region of interest (ROI) can be undertaken to evaluate the degree of spinal-stenosis [30]. Similar to MSCC, Fehlings et al. have developed a protocol to assess the maximum canal compromise (MCC) post-traumatic cervical spine-injury [69]. This has been additionally utilised for degenerative conditions and functions by calculating the canal size at the ROI and analysing it in conjunction to the average canal size for levels above and below. Multi-level signal-intensity changes are suggestive of necrosis or cavitation in the spinal cord and lend to poorer surgical outcomes [70,71,72,73]. T2-hyperintensity in conjunction with T1-hypointensity is associated with greater clinical deterioration when compared to T2-hyperintensity alone due to signal changes in T1-weighted images indicative of more permanent insult [70,74,75,76,77].

Limitations: Findings on conventional MRI do not typically correlate well with the variable clinical presentations of DCM [42]. Although spinal-cord compression is a sensitive marker of myelopathy [78], approximately 5% of asymptomatic patients also present with it [42], thereby limiting its specificity. The supine patient positioning in conventional MRI hinders its utility in assessing alignment, providing only a superficial assessment for situations in which upright films are not available [79]. Conventional MRI is intrinsically limited in its capability to characterize tissue injury in the spinal-cord because of the lack of specificity in T1/T2WI signal-change and cannot highlight specific pathophysiological processes at a cellular level (demyelination, axonal loss, inflammation, oedema, gliosis and apoptosis) [57]. It also is not a good predictor of neurologic function before/after surgical intervention and has low sensitivity for structural spinal cord change in cervical myelopathy [57,70,80,81,82,83].

#### 1.3.4. Plain Radiographs and Computed Tomography (CT)

Computed Tomography is useful for the study of bone anatomy and can aid in cases where spinal-fusion is being considered as a treatment. In cases where MRI is contraindicated (such as the presence of pacemakers or other internal metallic objects), CT is a valuable imaging alternative. Plain radiographs can provide useful information about spinal-canal stenosis, degenerating discs, degenerating joints, OPLL, vertebrae fusion, cervical-spine alignment and subluxation [2,38,84,85]. This can reveal scoliosis and loss of physiological cervical-lordosis and kyphosis. Lateral-films in cervical-flexion and extensions are utilised to evaluate instability of the cervical-spine. DCM patients frequently showcase increased C2-C7 Cobb angles, upper T1 slopes, lower C7 slopes and upper C7 slopes [86].

Limitations: Computed tomography suffers the same inability to characterise tissue injury that conventional MRI does [57]. In addition, a 2017 systematic-review found that the overall strength of evidence regarding the predictive value that CT parameters have for the clinical presentation or outcome of DCM is low [87]. There is also the issue of radiation exposure. Overall CT and plain radiographs play a more complementary role in DCM diagnosis, acting as an alternative to MRI and aiding in surgical-planning [88].

#### 1.3.5. Electrophysiology

Several studies have indicated good correlation between electrophysiology and the severity of myelopathy, presenting it as a reliable predictor of surgical-outcomes [89]. Somatosensory evoked-potentials (SEPs) and motor evoked-potentials (MEPs) can be, respectively, utilised to detect central sensory conduction impairment and prolonged motor latency in DCM [2,89,90]. They are also useful in detecting subclinical degenerative spinal-cord compression in asymptomatic patients and are thus useful in early identification of patients likely to develop myelopathy [91,92,93,94]. Feng et al. reported a correlation between the SEP and a declining mJOA (a more severe deficit) in an investigation of progressive myelopathy [95]. Needle electromyography (EMG) is a highly sensitive indicator of anterior horn cells damage, which occurs due to compression and ischemia in DCM [96]. Nerve-conduction studies can also be used to rule out peripheral neuropathy and nerve-entrapment [2]. These techniques also allow other neuromuscular diseases that can mimic DCM to be ruled out (motor neurone disease, ALS) [97,98]. Apart from aiding in diagnosis and preoperative evaluation, electrophysiology facilitates longitudinal assessment. Capone et al. found that a decrease in central-motor conduction time for the tibialis-anterior muscle correlated with an increased mJOA score post-surgery. It therefore concluded that the beneficial effects of spinal-cord surgery could be detected with MEP, making it a useful tool in determining efficacy of post-operative rehabilitation [99].

Limitations: Electrophysiology provides no anatomical information and thus cannot determine the exact location of the lesion [100]. Although some evidence exists to justify the effectiveness of electrophysiology in predicting operative outcomes, the area remains to be better defined. A systematic review found a decrease in electrophysiology publications compared with other domains of DCM, suggesting a declining interest in this area [101]. Additional studies would be required before it can be universally recommended.

### 1.4. Novel qMRI Modalities and Parameters

The limitations of current diagnostic options render DCM still largely a clinical diagnosis [17], making it necessary to develop and further research on novel diagnostic options with objective quantitative measures.

Advanced novel MRI protocols have been developed for the spinal cord that allow for acquisition within 45 min [102]. This involves direct measurement of spinal cord tissue changes, demyelination, axonal-injury and atrophy and thus renders the attainment of quantitative microstructural sequences now possible in the context of DCM. Such qMRI sequences and their derivable quantitative metrics are highlighted in Table 3. These derived metrics are highly sensitive to the myelopathic progression and can allow for the realisation of subclinical tissue-damage in patients with asymptomatic cervical-cord compression [103,104,105,106,107]. Quantitative metrics derived from DWI, such as DTT and DTI have been found to be more valuable when compared to conventional MRI scans in aiding diagnosis and outcome prediction in patients with DCM [108,109]. These qMRI sequences will be expanded upon later in this review. As an emerging field, the development of more advanced imaging techniques may potentiate in superior diagnostic tools, improved correlation with impairment and long-term predictions of DCM outcomes.

### 1.5. Objective

A scoping review was conducted in order to systematically map the research done in this area, as well as to identify any existing gaps in knowledge. The following research question was formulated:

*‘What is known from the literature about existing clinical and novel research MRI techniques for assessing spinal cord integrity in patients with Degenerative Cervical Myelopathy (DCM)?’*

## 2. Methodology

### 2.1. Data Sources

Articles published up to November 2021 were retrieved from three main databases: Medline, EMBASE and EBM. Combinations and variations of keywords were used to conduct a comprehensive search: spinal cord, spine, neck, magnetic resonance imaging, MRI, qMRI, T1, T2, T2*, R2*, DTI, diffusion tensor imaging, MT, magnetisation transfer, SWI, susceptibility weighted imaging BOLD, blood oxygen level dependent, fMRI, functional MRI, functional magnetic resonance imaging, MRS, magnetic resonance spectroscopy.

### 2.2. Selection Criteria

**Figure 3:**
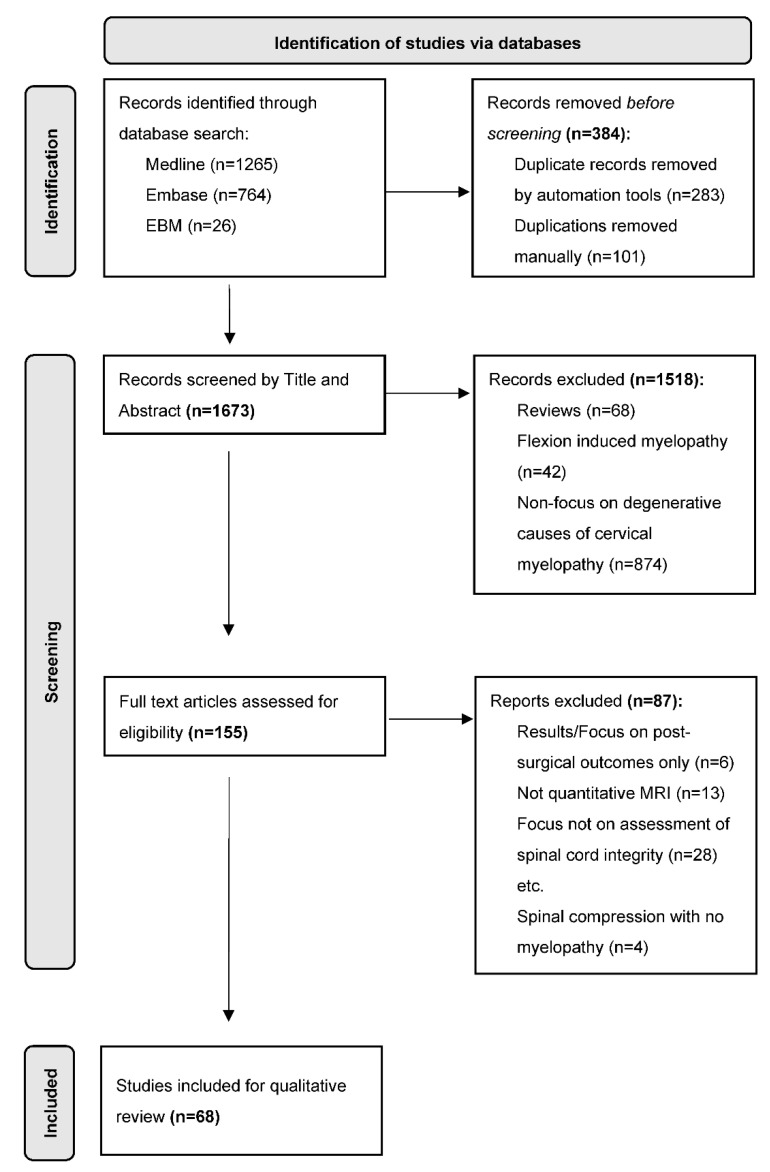
Literature Search Flowchart (See Appendix B—Figure A1. for database search strategy). Adapted from PRISMA Scoping Review protocol [121]. Abbreviations: MRI, magnetic resonance imaging.

Papers to be included required a focus on quantitative MRI techniques for assessing spinal cord integrity in patients with DCM. Exclusion criteria included any articles focusing on non-myelopathic diseases, flexion induced myelopathy, conventional MRI techniques only, non-degenerative causes of myelopathy, and studies on asymptomatic patients only. Reviews and non-English articles were also excluded from this study. A detailed flow diagram of this method can be seen in Figure 3.

### 2.3. Synthesis of Results

Following the process detailed in Figure 3., after studies were included for qualitative review detailed were collected in a tabular format (see Appendix C—Table A8). Details were then summarised (see Table 4 in Section 3) and underwent discussion and critical appraisal in Section 4.

## 3. Results

**Figure A1:**
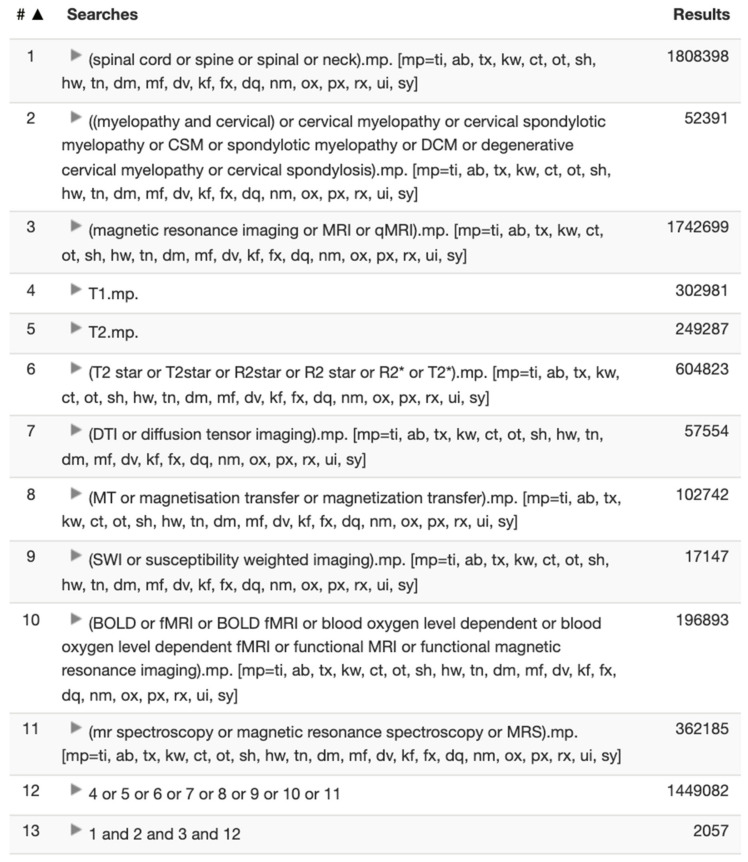
Database search strategy.

A total of 2055 articles were identified using the search strategy outlined in Figure 3 from the three databases. There were 283 duplicates removed by automation and 99 removed manually. The remaining 1770 records were then screened by title and abstract according to the inclusion and exclusion criteria leaving 155 articles to be screened for full-text eligibility. A detailed evaluation rendered 68 articles included for analysis. The complete database search strategy can be found in Appendix B (Figure A1).

## 4. Discussion

### 4.1. Quantitative T1 and T2 Mapping

#### 4.1.1. Principles

Quantitative T1/T2 mapping calculates the T1 (spin-lattice or longitudinal relaxation) time or T2 (spin-spin or transverse relaxation) time of a certain tissue and displays them on a parametric map. This is in contrast to conventional T1/T2-weighted MRI, which displays differences in T1/T2 relaxation times of tissues as a hyper/hypointense image with limited quantitative output [186,187]. T1 is influenced by the tissue’s water, lipid, protein and iron content which explains its sensitivity to pathological microstructural changes in the spinal cord [125,186]. T2 is influenced primarily by the water content of the tissue and thus increases in T2 is associated with increased water content pertaining to increased disc water or glycosaminoglycan content in the spinal-cord [187].

#### 4.1.2. Application in DCM

Despite being a fundamental parameter in MR imaging, T1/T2 mapping is not traditionally frequented in clinical practice due to its lengthy scan times. However, recent advances to speed of acquisition have enabled T1/T2 to become another viable option in the analysis of the pathological spinal cord [120,188,189,190,191]. Notably, T1 has been utilised in a multitude of brain studies to investigate changes in white and grey matter ratio in both a physiological and pathological context of which has demonstrated an interesting clinical correlation with cognitive decline [192,193]. Much of the literature surrounding its use in the spinal cord have highlighted reasonable scan times and reproducible methods to measure the T1 values in the healthy cervical spinal cord [120,194]. T2 relaxation times have been shown to correlate with lumbar disc degeneration [195,196,197], however our literature search reveals no studies conducted on the cervical spine. It thus pertains that T2 may potentiate as a useful biomarker for analysing spinal-cord integrity in DCM and so future research combining the two may prove beneficial.

From the conducted literature search, there exists only two studies applying T1 to the degenerative cervical spinal-cord which utilised 2D single section (inversion-recovery) radial-gradient echo [151] and 3D-MP2RAGE [125] sequences. The former study demonstrated that the difference in T1 relaxation times between stenotic segments and non-stenotic segments above and below were longer in higher grades of stenosis. It found that absolute T1 values were higher in high-grade stenosis but found inconclusive correlation between lower grades of stenosis [151]. The latter study utilising 3D-MP2RAGE found higher T1 values in the overall spinal-cord and the level of compression of patients with moderate-severe-DCM compared to healthy controls [125]. Overall, correlation findings of T1 and clinical severity are variable and inconclusive, particularly for patients with mild DCM which still maintain as the most difficult group to diagnose. Both studies are limited by their power and cross-sectional design. Further longitudinal studies with higher-sample sizes should be conducted particularly with respect to post-operative outcome to reach more conclusive results.

### 4.2. Diffusion Tensor Imaging (DTI)

#### 4.2.1. Principles

Diffusion tensor imaging is a sensitive measure of tissue microstructure that works by measuring the diffusion of water molecules. Water in a glass of water for example would be considered isotropic, the diffusion would be the same in every direction. The diffusion of water in tissue however would be considered anisotropic, whereby the diffusion will vary with direction. This would depend on the tissue type, integrity, architecture and presence of barriers. Fractional anisotropy (FA) (a quantifiable parameter of DTI) is a value between 0 and 1 that indicates the degree to which the diffusion of water is limited to one axis. Notably in a healthy spinal cord, the axons largely run in in one direction—commonly analogised to a bundle of straws. In DCM, the axonal integrity is compromised and by first principles, fractional anisotropy would be reduced. Other quantifiable DTI parameters include mean diffusivity (MD) or apparent diffusion coefficient which are both measures of the average magnitude of water diffusion within a tissue [102,118].

#### 4.2.2. Application in DCM

Recent studies have indicated a strong correlation between FA (from DTI) and clinical assessments such as mJOA [70,168,198,199,200]. Specifically Dong et al. found that DCM patients presenting with a higher FA score at the level of compression were the most likely group to achieve a better functional recovery after surgical decompression [199]. The conducted literature search indicated consistent findings of the correlation of FA and spinal cord integrity in DCM as well its significant role in prognosis [60,104,107,108,118,124,127,128,131,132,134,135,137,138,139,141,142,143,144,145,146,149,152,153,154,155,157,158,161,162,165,166,167,168,175,176,177,178,179,181,182,183,184,185]. Interestingly, Wang et al. conducted a prospective longitudinal study of 93 DCM patients and 36 healthy-controls and found that DTI parameter ratios (DTI measurement at test cervical level divided the measurement at C1-C2 levels) are more useful than absolute DTI metrics when assessing DCM as absolute metrics can be confounded by age and cervical level [108].

Therefore, DTI can be considered as not only a complementary diagnostic evaluation, but as a vital tool in the diagnosis of DCM and an early identifier of the candidates best suited to surgery. It is important to note however that as an emerging field, many existing studies [198,199,201,202,203] are of low-sample size and could suffer from potential bias from study design limits, patient selection bias or lack of blinding when examining new technologies. Studies utilising 1.5T-MRI as opposed to 3T-MRI are limited by poorer performance and resolution [158,201]. Additionally, some studies [134,158,177,183] are limited by their cross-sectional nature (limiting their ability to predict disease progression) and could benefit from more longitudinal components.

**Figure 4:**
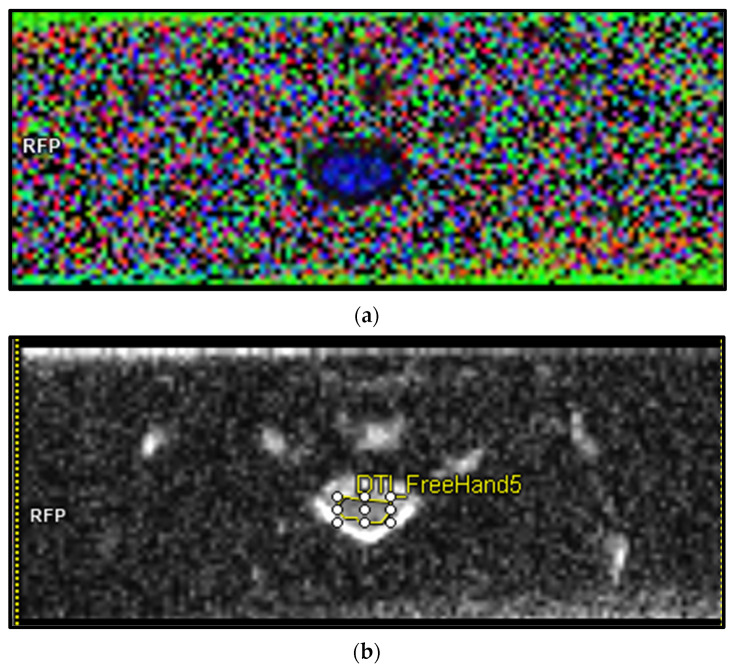
Preliminary data obtained from a healthy recruit using 3T MAGNETOM PRISMA MRI scanner with rFOV DTI ZOOMit sequence. From our standardisation work we have found rFOV to offer better visibility, better signal-to-noise, and less susceptibility and motion artifacts when compared to full field of view (fFOV) sequences. (**a**) axial Col-FA map of C4/5 cervical spinal-cord (red = left-right, blue = supra-inferior green = antero-posterior). (**b**) axial ADC map of C4/5 cervical spinal cord. Abbreviations: ADC, apparent diffusion coefficient; DTI, diffusion tensor imaging; Col-FA, colour fractional anisotropy; MRI, magnetic resonance imaging; rFOV, reduced field of view; ROI, region of interest.

From our conducted literature search, only 3/43 articles were of a prospective longitudinal design with a DCM cohort size of greater than 50. A total of 26/43 of the studies were of a cross-sectional design, and a majority of the longitudinal design studies had a short follow-up period of 3–6 months. As such there exists a need for additional large-scale longitudinal prospective studies to be conducted on DCM utilising DTI methods with longer time horizons and larger sample sizes. DCM is a chronic progressive disease and as it stands there exists no study that correlates longer-term progression (of over 3 years) with DTI parameter scores. Such quantifiable long-term studies could assist in identifying the characteristics of groups at risk of progressing deleteriously, thus contributing to the identification of patients who may benefit from early intervention. Further, in a prospective case control study, that slowed down due to COVID-19, we have standardised protocols (Table 5.) and successfully extracted data from healthy controls (Figure 4). This work will likely shed light on the spectrum of DCM when completed.

### 4.3. Functional MRI (BOLD)

#### 4.3.1. Principles

Functional-MRI (fMRI) is based on the BOLD contrast mechanism first introduced in 1990 and is a non-invasive technique that allows for the detection of neuronal activity. The fundamental principle behind the BOLD mechanism is that metabolic oxygen demand after neuronal activation causes a rise in blood flow and blood volume. This blood supply surpasses the actual oxygen needs which renders a transient rise of oxyhaemoglobin in the venous compartment and a relative decrease in the concentration of deoxyhaemoglobin. As deoxyhaemoglobin has paramagnetic properties, the change in deoxyhaemoglobin can be measured with MRI [112,204]. In DCM, fMRI has primarily been utilised to measure the functional connectivity (FC) and volume of activation (VOA) of regions of the brain before and after surgery to potentiate correlation [123,126,129,133,136,140,150,160,164,169,172,173,174,180].

#### 4.3.2. Application in DCM

The brain resting-state fMRI has been proven to be successful in differentiating DCM patients from healthy patients [123,126,150,173,174]. In contrast to the aforementioned MRI techniques which measures structural damage/integrity within the conduction pathways, BOLD fMRI measures the functional activation within the brain of which incorporates information collection, interpretation and distribution for all motor and cognitive functions. As a disease with a plethora of motor and sensory symptoms (see Table 1) DCM-associated information would be distributed to widespread areas of the brain [126,173]. As such, a multitude of studies have been performed to determine if fMRI can predict neurological recovery post-decompression surgery. Our literature search revealed that changes in FC strength between different areas of the brain appear to be associated with neurological improvement post-surgery in DCM. Numerous studies found an increase in VOA or FC strength of the pre/postcentral gyrus and SMA (supplementary motor area) following decompression surgery [123,126,133,140,164,174,180]. Functional connectivity alteration between the thalamus and cortex were also demonstrated [160]. Positive correlation of these findings with functional recovery assessed using various DCM grading questionnaires enables fMRI to indirectly assess spinal integrity in DCM patients.

Notably, Takenaka et al. found a positive correlation between post-operative improvement in the 10 s test (^[h]^ The 10 s test: the number of cycles the fingers can repeatedly grip and release in 10 s) and FC of three visual areas and the right superior-frontal gyrus in DCM patients, of which may enable the construction of a predictive formula for recovery potential [173]. Such a connection between visual cortices and DCM was also established in other studies [129,136]. Furthermore, an additional study by Takenaka et al. determined that resting-state amplitude of low-frequency fluctuation could function as a potential prognostic biomarker for DCM [172]. However, their two studies were limited by the use of mass univariate analyses which can only simply measure association. Given the multi-variable nature of fMRI, univariate analyses may miss information associated with DCM pathology and so studies using multivariate patten analysis should be conducted. Moreover, much of the research in the area is preliminary with studies of low sample sizes. Future external validation studies would be necessary for the proposed predictive formulas. None of the prospective studies have looked at the long-term use case of fMRI (over 6 months), thus development is also needed in this regard.

### 4.4. Magnetic Resonance Spectroscopy (MRS)

#### 4.4.1. Principles

MRS enables the in vivo quantification of metabolite concentration from human tissue. The underlying principle behind MRS is that a proton experiences a slightly distinct magnetic field of which is dependent on its chemical environment. Reliable quantification of metabolites utilising 1.5T MRI scanners have been traditionally limited to N-acetyllaspartate (NAA), choline (Cho) and creatine (Cr). However, recent advances in imaging technology and 3T MRI scanners have enabled measurement of glumatate-glumatine (Glx) and myo-inositols (Ins) [205]. N-acetylaspartate, despite not being a disease-specific marker is a sensitive indicator of axonal integrity due to its ability to be detected early in the disease course. Typically, NAA is expressed as an absolute value or as a ratio with Cho/Cr. The NAA/Cr ratio is generally viewed as a better ratio due to the more constant levels of Cr in the nervous system. However, changes in Cho are also believed to reflect increases in membrane phospholipids due to myelin breakdown from demyelinating diseases [113,118].

#### 4.4.2. Application in DCM

Cross-sectional studies have determined that the Cho/NAA ratio is higher in patients with DCM compared to healthy controls [171] and is significantly correlated with mJOA score [135,163]. As such it provides a potentially clinically useful biomarker for the management of DCM. Ellingson et al. utilised both DTI and MRS data in a combined linear model. The results of this optimised model showcased a higher accuracy in predicting mJOA than DTI and MRS alone [135]. Thus, MRS could find utility in tandem with DTI as a predictive tool. Interestingly, Kowalczyk et al. found that cortical levels of NAA/Cr could also serve as a meaningful biomarker in DCM [147,148]. Nagashima et al. investigated alternative metabolites (lactate, alanine, acetate, glutamate, pyruvate and citrate) and found no significant differences between the myelopathic and control group [156]. The main limitations in the area of MRS is that MR spectroscopic data within the spinal cord is quite difficult to acquire reliably due to patient motion, spinal cord movement (due to the pulsatile flow of CSF) and the difficulties associated with magnetic shimming (^[i]^ Magnetic shimming: the process by which the main magnetic field is made more homogenous) [135,147,148,156,163,171].

Overall, research of MRS application in DCM is quite limited with no new research being conducted in the last six years. Of the articles identified from our literature search, all were cross-sectional and of low-sample size. Further longitudinal work should be done to assess the prognostic potential of MRS in DCM.

### 4.5. Magnetisation Transfer (MT)

#### 4.5.1. Principles

Magnetisation transfer is a contrast mechanism that relies on the interaction between macromolecule bound hydrogen-protons, namely lipids and lipoproteins, and the free-protons (in free water) normally imaged by MRI. As such MT is able to indirectly probe proteins/lipids. The derived parameter, the magnetisation transfer ratio (MTR) reflects the portion of bound protons. Thus, MTR can be utilised as an indirect marker of demyelination and axonal loss as the MT effect indicates the relative density of protein/lipid macromolecules. This ability to measure myelin and axonal loss in vivo allows for application of MT to demyelinating diseases and degenerative diseases like DCM [115,118].

#### 4.5.2. Application in DCM

MTR has been well-established as a marker of myelin integrity in diseases such as multiple sclerosis [206] and has been shown to correlate with histopathological myelin loss [207]. MT imaging also presents an advantage over diffusion-based imaging in the form of higher signal-to-noise ratio and higher spatial resolution [170]. From our literature search, both Cloney et al. and Suleiman et al. found a negative correlation of MTR with severity of DCM (measured via the mJOA [130] and Nurick score [170], respectively), with pathological patients tending to have a decreased MTR compared with a healthy population. Such could be indicative of DCM associated myelin degradation [130,170]. However, Serbruyns et al. conducted a study that noted a decrease in MTR with aging [208]. The correlation of this with poorer functional tasks suggests that demyelination is associated with age-related decreases in functionality. As DCM is an elderly associated disease, this difficulty of determining causation means that MTR should be primarily interpreted as a quantitative measurement of demyelination from any cause, not just DCM. Paliwal et al. have also determined the prognostic potential of MTR for assessing response to surgery and recovery of DCM patients.

Perhaps the primary shortcoming of the current studies involves the small sample sizes and the limited number of prospective longitudinal studies. Future direction in this area could involve studies of higher sample sizes determining prognostic potential, utilisation of multivariate analysis as opposed to linear correlation, and longer follow up periods to track continued improvement beyond 6 months.

### 4.6. R2* or 1/T2*—A Promising Biomarker

#### 4.6.1. Principles

R2* MRI measures the ‘observable’ or ‘effective’ T2 (termed T2*) whereby $R2\;*=\frac{1}{T2*}$. T2* primarily results from inhomogeneities in the main magnetic field as a result of susceptibility-induced field distortion produced by the tissue placed within the field. In the presence of tissue iron, T2* relaxation time shortens and thus R2* increases (as $R2\;*=\frac{1}{T2*}$). Thus R2* represents a quantifiable measure of tissue iron content, notably via deoxyhaemoglobin, hemosiderin or methemoglobin in tissues and lesions [209,210].

#### 4.6.2. Role of Iron in Neurodegenerative Disorders

Homeostasis of heavy metals, such as iron and calcium are critical for cellular function. Imbalances in levels of iron and calcium have been implicated in various neurological disorders [211]. Iron plays an essential role in physiological functions during the ageing process. It is involved in DNA synthesis and repair, oxygen-transport, mitochondrial respiration, myelin synthesis, neurotransmitter synthesis and metabolism [212]. Abnormalities in homeostasis can induce oxidative damage through generation of reactive oxygen species and result in cellular death [212,213,214].

Past and present studies have indicated the disruption of iron homeostasis in a multitude of neurodegenerative diseases such as multiple sclerosis (MS) [215,216,217,218], Alzhiemer’s disease (AD) [219], Parkinson’s disease (PD) [220], Hallervorden-Spatz syndrome [221] and other pathologies involving iron accumulation in the brain [222]. Additionally, evidence indicates abnormal increases in calcium-signalling in AD [223], PD and amyotrophic lateral sclerosis (ALS) [224].

As a predominant neurodegenerative disorder of the ageing population; these changes in iron/calcium level could be implicated in DCM. As it stands; there is no current research in this area and studies looking to quantify these levels could assist in developing new diagnostic options and aid in understanding of the pathological processes of DCM at a molecular level.

#### 4.6.3. Application in DCM

A review of the literature revealed a plethora of studies that were successful in utilising R2*-MRI to quantify iron levels in brain for conditions such as AD [223,225,226,227], PD [228,229,230,231] and MS [232,233,234,235] in an effort to gauge correlation with the disease. A 2018 study utilised this R2*-MRI to quantify iron accumulation following acute traumatic spinal-cord injury [236] and found an increase in brain and brainstem iron accumulation following progressive neurodegeneration of patients. This study however did not explore iron accumulation in the spinal-cord. A 2013 study did however look into iron accumulation in the spinal-cord of mice following traumatic spinal-cord injury and in chronic stages post-injury, using MRI and histological techniques [237]. They were able to detect these iron deposits at the lesion site with live MRI and confirmation with Prussian-blue stains. There has not yet been a study that has looked into spinal-cord iron accumulation in non-traumatic DCM. As the most common cause of spinal-cord dysfunction, a study conducted in this area would prove to be beneficial in developing a new potential MRI biomarker for use in diagnosis.

**Figure 5:**
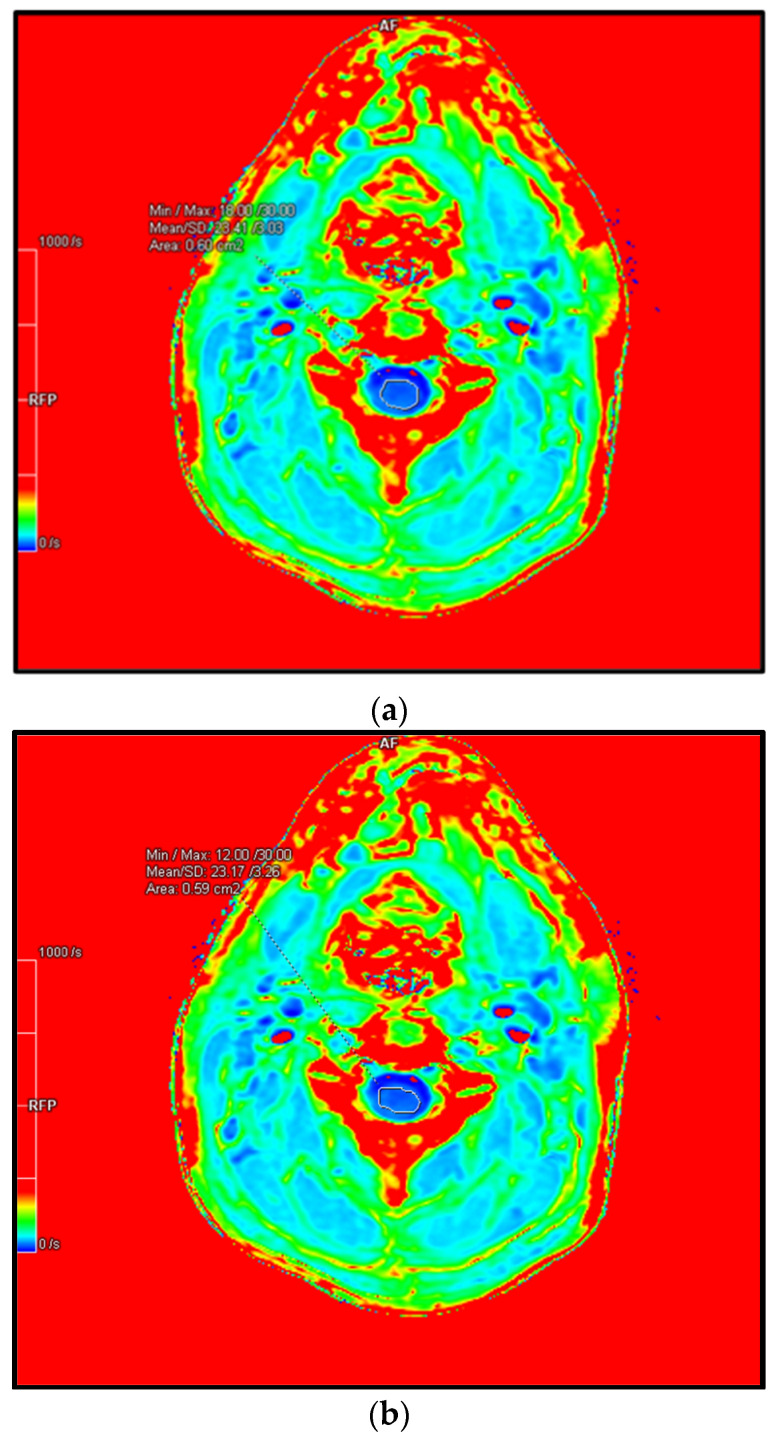

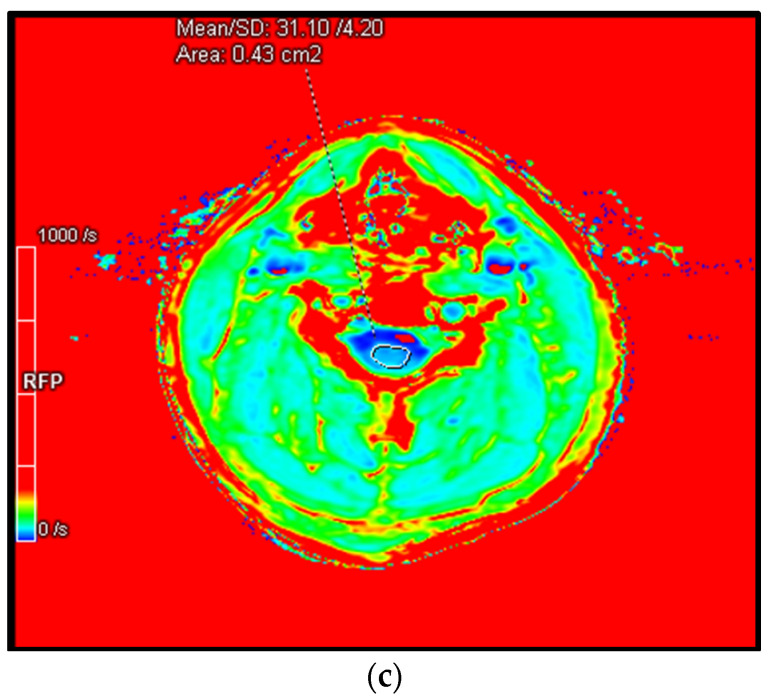
Preliminary data obtained from a healthy recruit using 3T MAGNETOM PRISMA MRI scanner with R2* MRI. (**a**) axial R2* map of C2/3 cervical spinal-cord. (**b**) axial R2* map of C4/5 cervical spinal-cord. (**c**) axial R2* map of C2/3 cervical spinal-cord. Abbreviations: DCM, degenerative cervical myelopathy; MRI, magnetic resonance imaging; ROI, region of interest.

In Table 6 and Figure 5 we present preliminary unpublished data of R2* ROI scores of the spinal cord of a healthy recruit. These data were obtained through our R2* MRI standardisation protocol and demonstrates the feasibility of this technique to be utilised in the spinal cord of patients. Further work must now be done with regard to DCM patients.

### 4.7. Quantitative Susceptibility Weighted Imaging (SWI)/Mapping—Another Promising Biomarker

#### 4.7.1. Underlying Principle

Compounds that have paramagnetic, ferromagnetic and diamagnetic properties all interact with the local magnetic field created by MRI. These compounds distort the local magnetic field and alter the phase of the tissue, which ultimately results in a change in signal. SWI is an MRI sequence that is particularly sensitive to such compounds and is therefore commonly used to detect blood products/haemorrhage and calcium. SWI utilises both the effect on phase and the magnitude, unlike conventional MRI sequences. After acquisition, post-processing involves the application of a high-pass filter that removes background inhomogeneity of the magnetic field and the employment of a phase mask which is used to accentuate the change in signal. This culminates in a susceptibility-weighted image which simultaneously incorporates magnitude and phase information for clinical use [238,239,240,241].

#### 4.7.2. Role of Calcium in Neurodegenerative Disorders

Calcium also plays an essential role in the ageing process. Physiological Ca^2+^ fluxes across plasma membranes and between intracellular compartments play vital roles in neuronal function such as in synaptic-transmission and plasticity, regulating neurite-growth and synaptogenesis, and cell survival. In neurodegenerative disorders these systems are compromised resulting in neuronal degeneration and dysfunction [242,243,244].

Interestingly, studies revealed the role of cellular iron in the stimulation of calcium signalling [245,246,247]. Whilst physiologically, this relationship assists in enhancing calcium-dependent signalling-pathways, an excessive iron accumulation promotes oxidative stress and a pathological upsurge in calcium-signals, of which results in mitochondrial damage. Moreover, this mitochondrial dysfunction renders a loss of iron homeostasis. If uncontrolled, this manifests a deleterious self-perpetuating cycle which eventuates in neuronal death.

#### 4.7.3. Application in DCM

The important self-inducive relationship between iron and calcium renders calcium an important area of research in the scope of neurodegenerative disorders such as DCM. Multiple studies have indicated calcium overload (calcification) at the impact site of acute traumatic spinal-cord injuries [248,249,250]. It has been yet to be determined whether calcium accumulation occurs during the course of DCM. Modern SWI is a MRI sequence that is particularly sensitive to compounds that distort local magnetic-fields and has been successfully utilised to measure calcium accumulation in the brain in vivo [238,251,252,253,254,255]. Of these studies includes a 2010 prospective study [255] with high-sample sizes and a varied population (age and gender). Extending protocols to image the spinal-cord could also prove beneficial in biomarker development.

## 5. Conclusions and Future Directions

For qMRI to attain clinical significance in DCM it must satisfy three overarching pillars of improvement. Firstly, the necessary advances must be made to minimise issues associated with artifacts and distortions whilst simultaneously improving on image quality, signal-noise ratio and spatial resolution. Such improvements will render qMRI both accurate and able to obtain repeatable results. Secondly, such advances must be utilised to further the literature on DCM, taking in account the limitations of current studies and inadequate areas of research as pointed out in this review. Finally, being both a novel and complex area of study, education is a priority, whereby researchers and clinicians must be updated on these novel quantitative techniques to enable more widespread and effective usage. This in turn will garner further research into this area. Notably, further longitudinal studies with higher sample sizes and longer time horizons are necessary to determine the full prognostic capacity of qMRI in DCM.

## Figures and Tables

**Table 1:** Typical presenting symptoms and physical signs in DCM [2,37,38,39,40,41,42,43].

	Presenting Symptoms	Physical Signs
Neck	– Pain and/or stiffness – Decreased cervical range of motion – The Lhermitte phenomenon ^[a]^	– Corticospinal tract distribution motor deficits
Upper Limb	– Weakness – Pain – Paraesthesia	– Upper motor neuron signs (hyper-reflexia, a positive Hoffman sign ^[b]^, a positive Trömner sign ^[c]^) – Sensory loss in a dermatomal pattern – Intrinsic hand muscle atrophy – Corticospinal tract distribution motor deficits
Lower Limb	– Weakness – Pain – Paraesthesia – Loss of manual dexterity – Falls – Gait imbalance	– Upper motor neuron signs (hyper-reflexia, a positive Babinski sign ^[d]^) – An unstable, broad-based gait – Sensory loss in a dermatomal pattern – Spasticity and clonus – Corticospinal tract distribution motor deficits
Urinary/defecatory	– Frequency/urgency – Urge incontinence	– Nil

Abbreviations: DCM, degenerative cervical myelopathy. [a] Lhermitte phenomenon: an electric shock-like sensation radiating from the neck down into the back that occurs upon flexion of the neck; [b] Positive Hoffman sign: flexion and adduction of the thumb and flexion of the index finger upon forceful flicking of the middle fingernail; [c] Positive Trömner sign: flexion of the thumb and index finger in response to flicking of the volar surface of the distal phalanx of the middle finger; [d] Positive Babinski sign: an upwards plantar response involving toe flexion after elicitation from the sole of the foot.

**Table 2:** Common classification systems used for DCM [17,47,48,49,50,51,52,53,54,55,56]. See Appendix A (Table A1, Table A2, Table A3, Table A4, Table A5, Table A6 and Table A7.) for full scoring systems.

System	Description	Benefits	Limitations
mJOA scale	– 0–18. A lower score indicates a more severe deficit. – Normal: 18 – Mild myelopathy: 15–17 – Mild myelopathy: 15–17 – Moderate myelopathy: 12–14 – Severe myelopathy: 0–11 – Upper-extremity function (5) – Lower-extremity function (7) – Sensory function (3) – Bladder function (3)	– Good for assessing outcomes (post-operative). – Specific to DCM – Responsive to change – Commonly used in research – Clinician administered	– No economic factors taken into consideration. – Poor sensitivity – Ceiling effect: hard to detect minor improvements in patients with mild disease – Modest intra-rater and inter-rater reliability (the minimum detectable change is two points). – Four categories are not equally weighted.
Nurick scale	– 0–5. A higher grade indicates a more severe deficit. – Myelopathy (6 points) – See Table A5. for grade definitions	– Good for evaluating economic situation in conjunction with gait function. – Specific to DCM – Commonly used in research – Consists of both impairment and disability components	– Low sensitivity – Poor responsiveness with limited ability to detect change. – Less accurate for post-operative grading. – Cannot detect upper extremity dysfunction.
NDI	– 0–50. A higher grade indicates a more severe disability. – Neck disability (10 subsections) – 0 = no disability5 = complete disability – Consists of: Pain intensity, personal care, lifting, reading, headaches, concentration, work, driving, sleeping, recreation	– Fair interobserver reliability in patients that have cervical radiculopathy – Responsive to change – Incorporates various activities from daily living	– Validity and reliability only evaluated in neck pain patients and cervical radiculopathy patients – Subjective; patient reported – Not specific to DCM
EMS	– 5–18. A lower score indicates a more severe deficit. – Normal: 17+ – Grade 1: 13–16 – Grade 2: 9–12 – Grade 3: 5–8	– Good at evaluating clinical state and level of severity. – Better sensitivity towards functional deficits (as it assesses coordination and proprioception)	– Not commonly used in research
CMS	– Upper/lower extremities are analysed separately 0–5 each. – A higher grade indicates a more severe deficit.	– Good for evaluating upper/lower extremity function as they are elicited separately. – Good at evaluating clinical state and level of severity.	– No economic factors taken into consideration.
NPRS	– 0–10. A higher score indicates a more severe disability	– Simplicity and reproducibility – Sensitive to small changes – Valid	– Not specific to DCM – Subjective – Suffers from the ceiling effect
EQ-5D	– A standardised measure of health status looking into mobility, self-care, activities of daily living, pain/discomfort, anxiety/depression. – Not measured on a numbered scale	– Ease of completion – Sensitive to change – Useful for looking into health economic evaluations	– Emotions and mood are limited to anxiety and depression – Quite global in nature – Overlooks some dimensions of quality of life (spiritual, social) – Does not include cognition – Not specific to DCM
Additional scales that provide useful information in the context of DCM include the Myelopathy Disability Index, QuickDASH (assesses arm, shoulder and hand disability), the 30-Metre-Walk test, the Berg Balance Scale, GAITRite (a temporospatial gait analysis) and the Graded Redefined Assessment of Strength Sensibility and Prehension Myelopathy (GRASSP-M).			

Abbreviations: CMS, Cervical Myelopathy Scale; DCM, degenerative cervical myelopathy; EMS, European Myelopathy Scale; mJOA, modified Japanese Orthopaedic Association; NDI, Neck Disability Index; NPRS, Numeric Pain Rating Scale.

**Table 3:** Quantitative MRI sequences applicable in the context of myelopathy and their corresponding derived metrics [70,103,104,105,106,107,110,111,112,113,114,115,116,117,118,119,120].

Sequence		Function	Quantitative Metrics
Quantitative T1/T2 Mapping		Calculates the T1/T2 time of certain tissues and displays them on a parametric map. Reveals information about microstructural changes related to water, lipid, protein and iron content of tissues.	T1/T2 relaxation time
DWI	DTI	Estimates the integrity of tissue microstructure through the modelling of water diffusion within the tissue.	FA ^[f]^, ADC, MD ^[g]^
	DTT	Tracks nerve fibres based on their FA values and can be elicited when fibres become interrupted, distorted or disorientated depending on the severity of spinal compression.	Volume and number of fibres
	DBSI	Quantifies axonal injury, inflammation and demyelination in DCM	Axonal injury, inflammation, demyelination.
fMRI (BOLD)		Measures neuronal activity through associated changes detected in blood flow	FC, VOA
MT		Provides information on the spinal cord structural integrity and derive information regarding myelination status	MTR
MRS		Sensitive to metabolic changes that occur in pathology, reflecting important underlying biological mechanisms	Metabolite concentrations
T2*-weighted imaging		Quantifies observable or effective T2 and is utilised to detect deoxyhaemoglobin, hemosiderin or methemoglobin in tissues and lesions.	R2* (=1/T2*)
SWI/QSM		Sensitive to compounds that distort the magnetic field and alter phase of tissue and is therefore commonly used to detect blood products/haemorrhage and calcium	Tissue susceptibility

Abbreviations: ADC, apparent diffusion coefficient; BOLD, blood oxygen level dependent; DBSI, diffusion basis spectrum imaging; DCM, degenerative cervical myelopathy; DTI, diffusion tensor imaging; DTT, diffusion tensor tractography; DWI, diffusion weighted imaging; FA, fractional anisotropy; FC, functional connectivity; fMRI, functional magnetic resonance imaging; MD, mean diffusivity; MRS, magnetic resonance spectroscopy; MT, magnetisation transfer; MTR, magnetisation transfer ratio; QSM, quantitative susceptibility mapping; SWI, susceptibility weighted imaging; VOA, volume of activation. ^[f]^ Fractional anisotropy (FA): Water molecules diffuse differently along tissues depending on its type, integrity, architecture, and presence of barriers. Fractional anisotropy is a value between 0 and 1 which indicates the degree to which diffusion of water is limited to one axis; ^[g]^ Apparent diffusion coefficient (ADC)/mean diffusivity (MD): measures of the average magnitude of water diffusion within a tissue.

**Table 4:** Summary of qMRI techniques utilised in the 68 included articles of this study (N.B. some studies investigated a multiplicity of qMRI techniques). Refer to Appendix C (Table A8) for included articles [60,104,105,108,122,123,124,125,126,127,128,129,130,131,132,133,134,135,136,137,138,139,140,141,142,143,144,145,146,147,148,149,150,151,152,153,154,155,156,157,158,159,160,161,162,163,164,165,166,167,168,169,170,171,172,173,174,175,176,177,178,179,180,181,182,183,184,185].

qMRI Technique Utilised	Number of Studies	Overall Findings from the Included Literature
Quantitative T1	2	– Higher T1 values in spinal cord of moderate-severe DCM – Inconclusive/variables results about mild cervical cord stenosis and mild DCM – Studies limited by low sample size and cross-sectional design
Quantitative T2	0	Nil
DTI	43	– Strong correlation of FA and mJOA – FA as a significant prognostic indicator – Need for more longitudinal large sample-size studies with longer time-horizons – DTI ratios as a better assessment metric than absolute DTI value.
fMRI (BOLD)	15	– Successful in differentiating DCM patients from healthy controls – Positive correlation of FC and VOA with various connections of the brain with post-surgical recovery – Notable correlation with visual cortices – Further external validation studies necessary – A need for prospective studies over 6 months to be conducted
MRS	6	– Cho/Naa ratio presents the best correlation with DCM severity. – Limitations with acquisition reliability – No new literature conducted in past 6 years – All cross-sectional and of low sample size – Further longitudinal and prognostic studies necessary
MT	4	– MTR negatively correlates with DCM severity – Potential confounding of data with MTR being additionally associated with age related demyelination – Low sample sizes – Overall limited research in this area, further longitudinal prospective studies required
R2* or 1/T2*	0	Nil
SWI	0	Nil

Abbreviations: BOLD, blood oxygen level dependent; Cho, choline; DCM, degenerative cervical myelopathy; DTI, diffusion tensor imaging; FA, fractional anisotropy; FC, functional connectivity; fMRI, functional magnetic resonance imaging; mJOA, modified Japanese Orthopaedic Association scale; MRS, magnetic resonance spectroscopy; MT, magnetisation transfer; MTR, magnetisation transfer ratio; NAA, n-acetylaspartate; qMRI, quantitative magnetic resonance imaging; SWI, susceptibility weighted imaging; VOA, volume of activation.

**Table 5:** Preliminary data obtained from our initial incomplete study, showcasing FA and ADC ROI scores of a healthy recruit. Data obtained through a standardisation protocol of DTI using 3T MAGNETOM PRISMA MRI scanner with a rFOV ZOOMit sequence with 4 averages (dynamic excitation for selective centrally excited field of view). We expect FA to be lower in recruits with DCM.

Measurements	Min/Max (×10^−3^)	Mean (×10^−3^)	Standard Deviation (×10^−3^)	Area (cm^2^)
FA	219/1000	629.16	201.72	0.35
ADC	186/1222	752.89	238.79	0.35

Abbreviations: ADC, apparent diffusion coefficient; DCM, degenerative cervical myelopathy; DTI, diffusion tensor imaging; FA, fractional anisotropy; MRI, magnetic resonance imaging; rFOV, reduced field of view; ROI, region of interest.

**Table 6:** Preliminary data obtained from our initial incomplete study, showcasing R2* region of interest (ROI) scores of a healthy recruit. Data obtained through a standardisation protocol of R2* using 3T MAGNETOM PRISMA MRI scanner. The values obtained from our R2* is equivalent to other soft tissues in the absence of pathological processes leading to iron deposition (based on R2* studies done in the brain and liver) which tends around 30. As such, we would expect higher R2* values in recruits with DCM.

Cervical Level	Min/Max (1/s)	Mean (1/s)	Standard Deviation	Area (cm^2^)
C2/3	18.00/30.00	23.41	3.03	0.60
C3/4	12.00/30.00	23.17	3.26	0.59
C4/5	18.00/44.00	31.40	4.20	0.43

Abbreviations: DCM, degenerative cervical myelopathy; MRI, magnetic resonance imaging; ROI, region of interest.

## Abbreviations

**1.5TMRI**	1.5 Tesla magnetic resonance imaging
**3TMRI**	3 Tesla magnetic resonance imaging
**AD**	Alzheimer’s disease
**ADC**	Apparent diffusion coefficient
**ALS**	Amyotrophic lateral sclerosis
**BOLD**	Blood oxygen level dependent
**Cho**	Choline
**CMS**	Cervical myelopathy scale
**CR**	Compression Ratio
**Cr**	Creatine
**CSM**	Cervical spondylotic myelopathy
**CT**	Computed tomography
**DBSI**	Diffusion basis spectrum imaging
**DCM**	Degenerative cervical myelopathy
**DNA**	Deoxyribonucleic acid
**DTI**	Diffusion tensor imaging
**DTT**	Diffusion tensor tractography
**DWI**	Diffusion weighted imaging
**EMG**	Electromyography
**EMS**	European myelopathy scale
**FA**	Fractional anisotropy
**FC**	Functional connectivity
**fFOV**	Full field of view
**fMRI**	Functional MRI
**Glx**	Glutamate-glutamine
**Ins**	Myo-inositols
**MCC**	Maximum canal compromise
**MEPs**	Motor evoked potentials
**mJOA**	Modified Japanese Orthopaedic Association scale
**MRI**	Magnetic resonance imaging
**MRS**	Magnetic resonance spectroscopy
**MS**	Multiple sclerosis
**MSCC**	Maximum spinal cord compression
**MT**	Magnetization transfer
**MTR**	Magnetization transfer ratio
**MWF**	Myelin water fraction
**NAA**	N-acetylaspartate
**NCS**	Nerve conduction studies
**NDI**	Neck disability index
**NPRS**	Numeric pain rating scale
**OPLL**	Ossification of the posterior longitudinal ligaments
**PD**	Parkinson’s disease
**qMRI**	Quantitative magnetic resonance imaging
**QSM**	Quantitative susceptibility mapping
**R2*MRI**	R2* magnetic resonance imaging
**rFOV**	Reduced field of view
**ROI**	Region of interest
**SMA**	Supplementary motor area
**SSEPs**	Somatosensory evoked potentials
**SWI**	Susceptibility weighted imaging
**T1WI**	T1 weighted imaging
**T2*WI**	T2*-weighted imaging
**T2WI**	T2 weighted imaging
**VOA**	Volume of activation

## Appendix A. Classification Systems for DCM

**Table A1:** Modified Japanese Orthopaedic Association (mJOA) Score [256]).

Modified Japanese Orthopaedic Association (mJOA) Score		
**Circle one**	**I. Motor dysfunction score of the upper extremities**	
0	Inability to move hands	
1	Inability to eat with a spoon but able to move hands	
2	Inability to button shirt but able to eat with a spoon	
3	Able to button shirt with great difficulty	
4	Able to button shirt with slight difficulty	
5	No dysfunction	
**Circle one**	**II. Motor dysfunction score of the lower extremities**	
0	Complete loss of motor and sensory function	
1	Sensory preservation without ability to move legs	
2	Able to move legs but unable to walk	
3	Able to walk on flat floor with a walking aid (i.e., cane or crutch)	
4	Able to walk up and/or down stairs with hand rail	
5	Moderate to significant lack of stability but able to walk up and/or down stairs without hand rail	
6	Mild lack of stability but walk unaided with smooth reciprocation	
7	No dysfunction	
**Circle one**	**III. Sensation**	
0	Complete loss of hand sensation	
1	Severe sensory loss or pain	
2	Mild sensory loss	
3	No sensory loss	
**Circle one**	**IV. Sphincter dysfunction**	
0	Inability to urinate voluntarily	
1	Marked difficulty with micturition	
2	Mild to moderate difficulty with micturition	
3	Normal micturition	
	Mild myelopathy	mJOA from 15 to 17
	Moderate myelopathy	mJOA from 12 to 14
	Severe myelopathy	mJOA from 0 to 11

**Table A2:** Numeric Pain Rating Scale (NPRS) [257].

Pain Numeric Rating Scale										
1. On a scale of 0 to 10, with 0 being no pain at all and 10 being the worst pain imaginable, how would you rate your pain RIGHT NOW.										
0	1	2	3	4	5	6	7	8	9	10
No Pain										Worst Pain Imaginable
2. On the same scale, how would you rate your USUAL level of pain during the last week.										
0	1	2	3	4	5	6	7	8	9	10
No Pain										Worst Pain Imaginable
3. On the same scale, how would you rate your BEST level of pain during the last week.										
0	1	2	3	4	5	6	7	8	9	10
No Pain										Worst Pain Imaginable
4. On the same scale, how would you rate your WORST level of pain during the last week.										
0	1	2	3	4	5	6	7	8	9	10
No Pain										Worst Pain Imaginable

**Table A3:** Neck Disability Index (NDI) [258].

Neck Disability Index
Please answer every section and **mark in each section only the one box that applies to you**.
**Section 1: Pain Intensity**
I have no pain at the moment
The pain is very mild at the moment
The pain is moderate at the moment
The pain is fairly severe at the moment
The pain is very severe at the moment
The pain is the worst imaginable at the moment
**Section 2: Personal Care (Washing, Dressing, etc.)**
I can look after myself normally without causing extra pain
I can look after myself normally but it causes extra pain
It is painful to look after myself and I am slow and careful
I need some help but can manage most of my personal care
I need help every day in most aspects of self care
I do not get dressed. I wash with difficulty and stay in bed
**Section 3: Lifting**
I can lift heavy weights without extra pain
I can lift heavy weights but it gives extra pain
Pain prevents me lifting heavy weights off the floor, but I can manage if they are conveniently placed, for example on a table
Pain prevents me from lifting heavy weights but I can manage light to medium weights if they are conveniently positioned
I can only lift very light weights
I cannot lift or carry anything
**Section 4: Reading**
I can read as much as I want to with no pain in my neck
I can read as much as I want to with slight pain in my neck
I can read as much as I want with moderate pain in my neck
I can’t read as much as I want because of moderate pain in my neck
I can hardly read at all because of severe pain in my neck
I cannot read at all
**Section 5: Headaches**
I have no headaches at all
I have slight headaches, which come infrequently
I have moderate headaches, which come infrequently
I have moderate headaches, which come frequently
I have severe headaches, which come frequently
I have headaches almost all the time
**Section 6: Concentration**
I can concentrate fully when I want to with no difficulty
I can concentrate fully when I want to with slight difficulty
I have a fair degree of difficulty in concentrating when I want to
I have a lot of difficulty in concentrating when I want to
I have a great deal of difficulty in concentrating when I want to
I cannot concentrate at all
**Section 7: Work**
I can do as much work as I want to
I can only do my usual work, but no more
I can do most of my usual work, but no more
I cannot do my usual work
I can hardly do any work at all
I can’t do any work at all
**Section 8: Driving**
I can drive my car without any neck pain
I can drive my car as long as I want with slight pain in my neck
I can drive my car as long as I want with moderate pain in my neck
I can’t drive my car as long as I want because of moderate pain in my neck
I can hardly drive at all because of severe pain in my neck
I can’t drive my car at all
**Section 9: Sleeping**
I have no trouble sleeping
My sleep is slightly disturbed (less than 1 h sleepless)
My sleep is mildly disturbed (1–2 h sleepless)
My sleep is moderately disturbed (2–3 h sleepless)
My sleep is greatly disturbed (3–5 h sleepless)
My sleep is completely disturbed (5–7 h sleepless)
**Section 10: Recreation**
I am able to engage in all my recreation activities with no neck pain at all
I am able to engage in all my recreation activities, with some pain in my neck
I am able to engage in most, but not all of my usual recreation activities because of pain in my neck
I am able to engage in a few of my usual recreation activities because of pain in my neck
I can hardly do any recreation activities because of pain in my neck
I can’t do any recreation activities at all
**Score:___/150 Transform to percentage score x 100 = %points**
**Scoring**: For each section the total possible score is 5: if the first statement is marked the section score = 0, if the last statement is marked it = 5. If all ten sections are completed the score is calculated as follows: Example: 16 (total scored)50 (total possible score) x 100 = 32%
If one section is missed or not applicable the score is calculated: Example: 16 (total scored) 45 (total possible score) x 100 = 35.5%
Minimum Detectable Change (90% confidence): 5 points or 10 %points

**Table A4:** EQ-5D [259].

EQ-5D
By placing a checkmark in one box in each group below, please indicate which statements best describe your own health state today.
**Mobility**
I have no problems in walking about
I have some problems in walking about
I am confined to bed
**Self-Care**
I have no problems with self-care
I have some problems washing or dressing myself
I am unable to wash or dress myself
Usual Activities (e.g., work, study, housework, family or leisure activities)
I have no problems with performing my usual activities
I have some problems with performing my usual activities
I am unable to perform my usual activities
**Pain/Discomfort**
I have no pain or discomfort
I have moderate pain or discomfort
I have extreme pain or discomfort
**Anxiety/Depression**
I am not anxious or depressed
I am moderately anxious or depressed
I am extremely anxious or depressed

**Table A5:** Nurick Grading System [260].

	Nurick Grading System
Grade.	Definition
0	Signs or symptoms of root involvement, but without evidence of spinal cord disease.
I	Signs of spinal cord disease, but no walking difficulty.
II	Slight difficulty in walking that does not prevent full- time employment.
III	Walking difficulty that prevents full-time employment or the ability to do all housework but is not so severe as to require help from another person to ambulate.
IV	Able to walk only with help from another person or with the aid of a frame.
V	Bedridden or chairbound.

**Table A6:** European Myelopathy Score [49].

European Myelopathy Score		
Upper motor neuron		
1	Unable to walk, wheelchair	
Gait function		
2	Walking on a flat ground only with cane or aid	
3	Climbing stairs only with aid	
4	Gait clumsy, but no aid necessary	
5	Normal walking and climbing stairs	
**Upper motor neuron**		
1	Retention, no control over bladder and/or bowel function	
Bladder and bowel function		
2	Inadequate micturition and urinary frequency	
3	Normal bladder and bowel function	
**Lower motor neuron**		
1	Handwriting and eating with knife and fork impossible	
Hand function		
2	Handwriting and eating with knife and fork impaired	
3	Handwriting, tying shoe laces or a tie clumsy	
4	Normal handwriting	
**Posterior column**		
1	Getting dressed only with aid	
Proprioception and coordination		
2	Getting dressed clumsily and slowly	
3	Getting dressed normally	
**Paraesthesia/pain**		
1	Invalidity due to pain	
2	Endurable paraesthesia and pain	
3	No paraesthesia and pain	
	Normal function	17–18
	Grade 1	13–16
	Grade 2	9–12
	Grade 3	5–8

**Table A7:** Cooper Myelopathy Scale [261].

**Cooper Myelopathy Scale**	
**Upper extremity function**	
Grade 0	Intact
Grade 1	Sensory symptoms only
Grade 2	Mild motor deficit with some functional impairment
Grade 3	Major functional impairment in at least one upper extremity but upper extremities useful for simple tasks
Grade 4	No movement or flicker of movement in upper extremities; no useful function
**Lower extremity function**	
Grade 0	Intact
Grade 1	Walks independently but not normally
Grade 2	Walks but needs cane or walker
Grade 3	Stands but cannot walk
Grade 4	Slight movement but cannot walk or stand
Grade 5	Paralysis

## Appendix B. Database Search Strategy

**EBM Reviews—ACP Journal Club** 1991 to November 2021
**Embase** 1974 to 3 December 2021
**MEDLINE(R) All including Epub Ahead of Print, In-Process and Other Non-Indexed Citations, Daily and Versions(R)** 1946-current

## Appendix C. Article Study Characteristics

**Table A8:** Study characteristics of articles deemed eligible for inclusion by search strategy [60,104,105,108,122,123,124,125,126,127,128,129,130,131,132,133,134,135,137,138,139,140,141,142,143,144,145,146,147,148,149,150,151,152,153,154,155,156,157,158,159,160,161,162,163,164,165,166,167,168,169,170,171,172,173,174,175,176,177,178,179,180,181,183,184,185,187,188].

No.	Author(s)	Year	Title	Study Design	Follow-Up Period (Months)	Subjects	qMRI Technique	qMRI Parameters Tested
1	Maki, Satoshi; Koda, Masao; Kitamura, Mitsuhiro; Inada, Taigo; Kamiya, Koshiro; Ota, Mitsutoshi; Iijima, Yasushi; Saito, Junya; Masuda, Yoshitada; Matsumoto, Koji; Kojima, Masatoshi; Obata, Takayuki; Takahashi, Kazuhisa; Yamazaki, Masashi; Furuya, Takeo	2017	Diffusion tensor imaging can predict surgical outcomes of patients with cervical compression myelopathy	Prospective Longitudinal	6	DCM = 26	DTI	FA, MD
2	Bhosale, Sunil; Ingale, Pramod; Srivastava, Sudhir; Marathe, Nandan; Bhide, Prajakta	2019	Diffusion tensor imaging as an additional postoperative prognostic predictor factor in cervical myelopathy patients: An observational study	Prospective Longitudinal	3	DCM = 30	DTI	FA, MD
3	Song, Ting; Chen, Wen-Jun; Huang, Jian-Wei; Cai, Ming-Jin; Dong, Tian-Fa; Li, Tang-Sheng; Yang, Bo; Zhao, Hong-Pu	2011	Diffusion tensor imaging in the cervical spinal cord	Prospective Longitudinal	6	DCM = 53 Healthy Controls = 20	DTI	FA, ADC
4	Severino, Rocco; Nouri, Aria; Tessitore, Enrico	2020	Degenerative cervical myelopathy: How to identify the best responders to surgery?	Prospective Longitudinal	12	DCM = 36	DTI	FA
5	Nukala, Monika; Abraham, Jini; Khandige, Ganesh; Shetty, Bharath K.; Rao, Arindam pol arjun	2019	Efficacy of diffusion tensor imaging in identification of degenerative cervical spondylotic myelopathy	Prospective Cross-sectional	N/A	DCM = 50	DTI	FA, ADC
6	Ulubaba, Hilal Er; Saglik, Semih; Yildirim, Ismail Okan; Durak, Mehmet Akif	2021	Effectiveness of Diffusion Tensor Imaging in Determining Cervical Spondylotic Myelopathy	Prospective Cross-sectional	N/A	DCM = 54	DTI	FA, ADC
7	Tian, Xiaonan; Zhang, Li; Zhang, Xuesong; Meng, Linghui; Li, Xiaona	2021	Correlations between preoperative diffusion tensor imaging and surgical outcome in patients with cervical spondylotic myelopathy	Retrospective Longitudinal	12	DCM = 95	DTI	FA, ADC
8	Iwasaki, Motoyuki; Yokohama, Takumi; Oura, Daisuke; Furuya, Shou; Niiya, Yoshimasa; Okuaki, Tomoyuki	2019	Decreased Value of Highly Accurate Fractional Anisotropy Using 3-Tesla ZOOM Diffusion Tensor Imaging After Decompressive Surgery in Patients with Cervical Spondylotic Myelopathy: Aligned Fibers Effect	Prospective Longitudinal	6	DCM = 26Healthy Controls = 12	DTI	FA
9	Toktas, Zafer Orkun; Kilic, Turker; Konya, Deniz; Tanrikulu, Bahattin; Koban, Orkun	2016	Diffusion tensor imaging of cervical spinal cord: A quantitative diagnostic tool in cervical spondylotic myelopathy	Prospective Cross-sectional	N/A	DCM = 21	DTI	FA, ADC
10	Ellingson, Benjamin M.; Salamon, Noriko; Grinstead, John W.; Holly, Langston T.	2014	Diffusion tensor imaging predicts functional impairment in mild-to-moderate cervical spondylotic myelopathy	Prospective Cross-sectional	N/A	DCM = 48Healthy Controls = 9	DTI	FA, ADC, MD
11	Han, X.; Ma, X.; Li, D.; Wang, J.; Jiang, W.; Cheng, X.; Li, G.; Guo, H.; Tian, W.	2020	The Evaluation and Prediction of Laminoplasty Surgery Outcome in Patients with Degenerative Cervical Myelopathy Using Diffusion Tensor MRI	Prospective Longitudinal	6	DCM = 55Healthy Controls = 20	DTI	FA, MD
12	Guo, Xing; Yang, Xiaotian; Chen, Xukang; Zhao, Rui; Song, Yingchao; Liang, Meng; Sun, Haoran; Xue, Yuan	2021	Enhanced Information Flow From Cerebellum to Secondary Visual Cortices Leads to Better Surgery Outcome in Degenerative Cervical Myelopathy Patients: A Stochastic Dynamic Causal Modeling Study With Functional Magnetic Resonance Imaging	Prospective Longitudinal	6	DCM = 27Healthy Controls = 11	fMRI (BOLD)	Effective connectivity (EC)
13	Rajasekaran, S.; Kanna, Rishi M.; Chittode, Vishnuprasath S.; Maheswaran, Anupama; Aiyer, Siddharth N.; Shetty, Ajoy P.	2017	Efficacy of Diffusion Tensor Imaging Indices in Assessing Postoperative Neural Recovery in Cervical Spondylotic Myelopathy	Prospective Longitudinal	12	DCM = 26	DTI	ADC
14	Liu, Xiaojia; Qian, Wenshu; Jin, Richu; Li, Xiang; Luk, Keith Dk; Wu, Ed X.; Hu, Yong	2016	Amplitude of Low Frequency Fluctuation (ALFF) in the Cervical Spinal Cord with Stenosis: A Resting State fMRI Study	Prospective Cross-sectional	N/A	DCM = 18Healthy Controls = 25	fMRI (BOLD)	Amplitude of low frequency fluctuation (ALFF)
15	Cui, Jiao-Long; Li, Xiang; Chan, Tin-Yan; Mak, Kin-Cheung; Luk, Keith Dip-Kei; Hu, Yong	2015	Quantitative assessment of column-specific degeneration in cervical spondylotic myelopathy based on diffusion tensor tractography	Prospective Cross-sectional	N/A	DCM = 23Healthy Controls = 20	DTI	FA, MD
16	Nischal, Neha; Tripathi, Shalini; Singh, Jatinder Pal	2020	Quantitative Evaluation of the Diffusion Tensor Imaging Matrix Parameters and the Subsequent Correlation with the Clinical Assessment of Disease Severity in Cervical Spondylotic Myelopathy	Prospective Cross-sectional	N/A	DCM = 52	DTI	FA, ADC
17	Peng, Xinji; Tan, Yongming; He, Laichang; Ou, Yangtao	2020	Alterations of functional connectivity between thalamus and cortex before and after decompression in cervical spondylotic myelopathy patients: A resting-state functional MRI study	Prospective Longitudinal	3	DCM = 43Healthy Controls = 43	fMRI (BOLD)	BOLD signal
18	Tan, Yongming; Zhou, Fuqing; Liu, Zhili; Wu, Lin; Zeng, Xianjun; Gong, Honghan; He, Laichang	2016	Alteration of cerebral regional homogeneity within sensorimotor network in patients with cervical spondylotic myelopathy after spinal cord decompression: a resting-state functional MRI study	Prospective Longitudinal	3	DCM = 21Healthy Controls = 21	fMRI (BOLD)	Regional homogeneity (ReHo)
19	Kowalczyk, Izabela; Bartha, Robert; Duggal, Neil	2012	Proton magnetic resonance spectroscopy of the motor cortex in cervical myelopathy	Prospective Cross-sectional	N/A	DCM = 24Healthy Controls = 11	MRS	N-acetylaspartate/creatine
20	Lee, Seungbo; Chung, Tae-Sub; Kim, Sungjun; Yoo, Yeon Hwa; Yoon, Choon-Sik; Lee, Young Han; Suh, Jin-Suck; Jeong, Eun-Kee; Kim, In Seong; Park, Jung Hyun	2015	Accuracy of diffusion tensor imaging for diagnosing cervical spondylotic myelopathy in patients showing spinal cord compression	Prospective Cross-sectional	N/A	DCM = 33	DTI	FA, MD
21	Wang, K.Y.; Idowu, O.; Orman, G.; Izbudak, I.; Thompson, C.B.; Myers, C.; Riley, L.H.; Carrino, J.A.; Flammang, A.; Gilson, W.; Sadowsky, C.L.	2017	Tract-Specific Diffusion Tensor Imaging in Cervical Spondylotic Myelopathy Before and After Decompressive Spinal Surgery: Preliminary Results	Prospective Longitudinal	6	DCM = 4Healthy Controls = 5	DTI	FA, MD
22	Shabani, Saman; Kaushal, Mayank; Budde, Matthew; Schmit, Brian; Wang, Marjorie C.; Kurpad, Shekar	2019	Comparison between quantitative measurements of diffusion tensor imaging and T2 signal intensity in a large series of cervical spondylotic myelopathy patients for assessment of disease severity and prognostication of recovery	Prospective Longitudinal	24	DCM = 46	DTI	FA
23	Duggal, N.; Rabin, D.; Bartha, R.; Barry, R.L.; Gati, J.S.; Kowalczyk, I.; Fink, M.	2010	Brain reorganization in patients with spinal cord compression evaluated using fMRI	Prospective Longitudinal	6	DCM = 12Healthy Controls = 10	fMRI (BOLD)	Volume of Activation (VOA)
24	Jurova, Barbora; Mechl, Marek; Kerkovsky, Milos; Sprlakova-Pukova, Andrea; Kadanka, Zdenek; Nemec, Martin; Bednarik, Josef; Kovalova, Ivana; Dusek, Ladislav	2017	Spinal Cord MR Diffusion Properties in Patients with Degenerative Cervical Cord Compression	Prospective Cross-sectional	N/A	DCM = 130Healthy Controls = 71	DTI	FA, ADC
25	Kara, Batuhan; Celik, Azim; Karadereler, Selhan; Ulusoy, Levent; Ganiyusufoglu, Kursat; Onat, Levent; Mutlu, Ayhan; Ornek, Ibrahim; Sirvanci, Mustafa; Hamzaoglu, Azmi	2011	The role of DTI in early detection of cervical spondylotic myelopathy: a preliminary study with 3-T MRI	Prospective Cross-sectional	N/A	DCM = 16	DTI	FA, ADC
26	Maki, Satoshi; Koda, Masao; Ota, Mitsutoshi; Oikawa, Yoshihiro; Kamiya, Koshiro; Inada, Taigo; Furuya, Takeo; Takahashi, Kazuhisa; Masuda, Yoshitada; Matsumoto, Koji; Kojima, Masatoshi; Obata, Takayuki; Yamazaki, Masashi	2018	Reduced Field-of-View Diffusion Tensor Imaging of the Spinal Cord Shows Motor Dysfunction of the Lower Extremities in Patients with Cervical Compression Myelopathy	Prospective Cross-sectional	N/A	DCM = 20Healthy Controls = 10	DTI	FA
27	Hassan, Talaat Ahmed Abd El Hameed; Assad, Ramy Edward; Belal, Shaimaa Atef	2019	MR diffusion tensor imaging of the spinal cord: can it help in early detection of cervical spondylotic myelopathy and assessment of its severity?	Prospective Cross-sectional	N/A	DCM = 30	DTI	FA
28	Cloney, Michael Brendan; Smith, Zachary A.; Weber, Kenneth A.; Parrish, Todd B.	2018	Quantitative Magnetization Transfer MRI Measurements of the Anterior Spinal Cord Region are Associated with Clinical Outcomes in Cervical Spondylotic Myelopathy	Prospective Cross-sectional	N/A	DCM = 7Healthy Controls = 7	MT	MTR
29	Salamon, Noriko; Woodworth, Davis C.; Holly, Langston T.; Ellingson, Benjamin M.	2018	Resting-State Functional Magnetic Resonance Imaging Connectivity of the Brain Is Associated with Altered Sensorimotor Function in Patients with Cervical Spondylosis	Prospective Cross-sectional	N/A	DCM = 24Healthy Controls = 17	fMRI (BOLD)	Functional Connectivity (FC)
30	Wang, Chencai; Salamon, Noriko; Laiwalla, Azim; Holly, Langston T.; Ellingson, Benjamin M.; Islam, Sabah	2021	Supraspinal functional and structural plasticity in patients undergoing surgery for degenerative cervical myelopathy	Prospective Longitudinal	3	DCM = 19Healthy Controls = 16	fMRI (BOLD)	Functional Connectivity (FC)
31	Baucher, G.; Rasoanandrianina, H.; Levy, S.; Pini, L.; Troude, L.; Roche, P. H.; Callot, V.	2021	T1 Mapping for Microstructural Assessment of the Cervical Spinal Cord in the Evaluation of Patients with Degenerative Cervical Myelopathy	Prospective Cross-sectional	N/A	DCM = 20Healthy Controls = 10	Quantitative T1	T1
32	Banaszek, Anna; Bladowska, Joanna; Szewczyk, Pawel; Podgorski, Przemyslaw; Sasiadek, Marek	2014	Usefulness of diffusion tensor MR imaging in the assessment of intramedullary changes of the cervical spinal cord in different stages of degenerative spine disease	Prospective Cross-sectional	N/A	DCM = 132Healthy Controls = 25	DTI	FA, ADC
33	Ellingson, Benjamin M.; Salamon, Noriko; Hardy, Anthony J.; Holly, Langston T.	2015	Prediction of Neurological Impairment in Cervical Spondylotic Myelopathy using a Combination of Diffusion MRI and Proton MR Spectroscopy	Prospective Cross-sectional	N/A	DCM = 27Healthy Controls = 11	DTI, MRS	FA, MD, Cho/NAA (Choline/N-acetylaspartate)
34	Salamon, N.; Ellingson, B.M.; Nagarajan, R.; Gebara, N.; Thomas, A.; Holly, L.T.	2013	Proton magnetic resonance spectroscopy of human cervical spondylosis at 3T	Prospective Cross-sectional	N/A	DCM = 21Healthy Controls = 11	MRS	NAA (N-acetylaspartate), Cho (choline), Myo-I (myo-inositol) ratio with Cr (creatine)
35	Chen, Zhao; Zhao, Rui; Wang, Qiu; Yu, Chunshui; Li, Fengtan; Liang, Meng; Zong, Yaqi; Zhao, Ying; Xiong, Wuyi; Su, Zhe; Xue, Yuan	2020	Functional Connectivity Changes of the Visual Cortex in the Cervical Spondylotic Myelopathy Patients: A Resting-State fMRI Study	Prospective Longitudinal	3	DCM = 30Healthy Controls = 20	fMRI (BOLD)	Functional Connectivity (FC)
36	Bhagavatula, Indira Devi; Shukla, Dhaval; Sadashiva, Nishanth; Saligoudar, Praveen; Prasad, Chandrajit; Bhat, Dhananjaya I.	2016	Functional cortical reorganization in cases of cervical spondylotic myelopathy and changes associated with surgery	Prospective Longitudinal	6	DCM = 17Healthy Controls = 12	fMRI (BOLD)	Volume of Activation (VOA)
37	Murphy, Rory K.; Sun, Peng; Han, Rowland H.; Griffin, Kim J.; Wagner, Joanne; Yarbrough, Chester K.; Wright, Neill M.; Dorward, Ian G.; Riew, K. Daniel; Kelly, Michael P.; Santiago, Paul; Zebala, Lukas P.; Trinkaus, Kathryn; Ray, Wilson Z.; Song, Sheng-Kwei	2018	Fractional anisotropy to quantify cervical spondylotic myelopathy severity	Prospective Cross-sectional	N/A	DCM = 14Healthy Controls = 7	DTI	FA
38	Takenaka, Shota; Kan, Shigeyuki; Seymour, Ben; Makino, Takahiro; Sakai, Yusuke; Kushioka, Junichi; Tanaka, Hisashi; Watanabe, Yoshiyuki; Shibata, Masahiko; Yoshikawa, Hideki; Kaito, Takashi	2020	Resting-state Amplitude of Low-frequency Fluctuation is a Potentially Useful Prognostic Functional Biomarker in Cervical Myelopathy	Prospective Longitudinal	6	DCM = 28Healthy Controls = 28	fMRI (BOLD)	Amplitude of low frequency fluctuation (ALFF)
39	Cui, Libin; Chen, Xueming; Liu, Yadong; Zhang, Yanjun; Kong, Chao; Guan, Yun	2019	Changes in diffusion tensor imaging indices of the lumbosacral enlargement correlate with cervical spinal cord changes and clinical assessment in patients with cervical spondylotic myelopathy	Prospective Cross-sectional	N/A	DCM = 40Healthy Controls = 42	DTI	FA, ADC
40	Holly, Langston T.; Wang, Chencai; Salamon, Noriko; Woodworth, Davis C.; Ellingson, Benjamin M.	2019	Neck disability in patients with cervical spondylosis is associated with altered brain functional connectivity	Prospective Cross-sectional	N/A	DCM = 36Healthy Controls = 17	fMRI (BOLD)	Functional Connectivity (FC)
41	Grabher, Patrick; David, Gergely; Mohammadi, Siawoosh; Freund, Patrick	2017	Neurodegeneration in the Spinal Ventral Horn Prior to Motor Impairment in Cervical Spondylotic Myelopathy	Prospective Cross-sectional	N/A	DCM = 20Healthy Controls = 18	DTI	MD
42	Kerkovsky, M.; Jakubcova, B.; Mechl, M.; Kadanka, Z.; Kadanka Jr, Z.; Nemec, M.; Kovalova, I.; Bednarik, J.	2015	Multifactorial determination of the spinal cord diffusion properties in patients with cervical spondylotic spinal cord compression: A diffusion tensor imaging study	Prospective Cross-sectional	N/A	DCM = 130Healthy Controls = 71	DTI	FA, ADC
43	Kowalczyk, I.; Bartha, R.; Duggal, N.	2010	Proton magnetic resonance spectroscopy of the motor cortex in cervical spondylotic myelopathy	Prospective Cross-sectional	N/A	DCM = 24Healthy Controls = 11	MRS	NAA/Cr (N-acetylaspartate/creatine metabolite ratio)
44	Taha Ali, Tamer F.; Badawy, Ahmed E.	2013	Feasibility of 1H-MR Spectroscopy in evaluation of cervical spondylotic myelopathy	Prospective Cross-sectional	N/A	DCM = 34Healthy Controls = 11	MRS	NAA/Cr (N-acetylaspartate/creatine metabolite ratio), Cho/Cr (Chloline/creatine ratio)
45	Aleksanderek, Izabela K.; Stevens, Todd; Goncalves, Sandy; Bartha, Robert; Duggal, Neil	2017	Metabolite and functional profile of patients with cervical spondylotic myelopathy	Prospective Longitudinal	6	DCM = 28Healthy Controls = 10	fMRI (BOLD), MRS	Volume of Activation (VOA), NAA/Cr (N-acetylaspartate/creatine metabolite ratio)
46	Wen, Chun Yi; Cui, Jiao Long; Liu, Harris S.; Mak, Kin Cheung; Cheung, Wai Yuen; Luk, Keith D.K.; Hu, Yong	2014	Is diffusion anisotropy a biomarker for disease severity and surgical prognosis of cervical spondylotic myelopathy	Prospective Longitudinal	6 to 24	DCM = 45Healthy Controls = 20	DTI	FA
47	Paliwal, Monica; Smith, Zachary A.; Weber, Kenneth A.; Mackey, Sean; Hopkins, Benjamin S.; Dahdaleh, Nader S.; Cantrell, Donald R.; Parrish, Todd D.; Hoggarth, Mark A.; Elliott, James M.; Dhaher, Yasin	2020	Magnetization Transfer Ratio and Morphometrics of the Spinal Cord Associates with Surgical Recovery in Patients with Degenerative Cervical Myelopathy	Prospective Longitudinal	6	DCM = 13Healthy Controls = 9	MT	MTR
48	Martin, Allan R.; De Leener, Benjamin; Cohen-Adad, Julien; Kalsi-Ryan, Sukhvinder; Cadotte, David W.; Wilson, Jefferson R.; Tetreault, Lindsay; Nouri, Aria; Crawley, Adrian; Mikulis, David J.; Ginsberg, Howard; Massicotte, Eric M.; Fehlings, Michael G.	2018	Monitoring for myelopathic progression with multiparametric quantitative MRI	Prospective Longitudinal	12	DCM = 26	DTI, MT	FA, MTR
49	Chen, Xueming; Kong, Chao; Feng, Shiqing; Guan, Hua; Yu, Zhenshan; Cui, Libin; Wang, Yanhui	2016	Magnetic resonance diffusion tensor imaging of cervical spinal cord and lumbosacral enlargement in patients with cervical spondylotic myelopathy	Prospective Cross-sectional	N/A	DCM = 10Healthy Controls = 10	DTI	FA, ADC
50	Suleiman, Linda I.; Rosenthal, Brett D.; Bhatt, Surabhi A.; Hsu, Wellington K.; Patel, Alpesh A.; Parrish, Todd B.; Savage, Jason W.; Weber, Kenneth A.	2018	High-resolution magnetization transfer MRI in patients with cervical spondylotic myelopathy	Prospective Cross-sectional	N/A	DCM = 10Healthy Controls = 7	MT	MTR
51	Nagashima, Hideki; Nanjo, Yoshiro; Teshima, Ryota; Morio, Yasuo; Meshitsuka, Shunsuke; Yamane, Koji	2010	High-resolution nuclear magnetic resonance spectroscopic study of metabolites in the cerebrospinal fluid of patients with cervical myelopathy and lumbar radiculopathy	Prospective Cross-sectional	N/A	DCM = 30Healthy Controls = 10	MRS	Lactate, alanine, acetate, glutamate, pyruvate, citrate
52	Su, Qian; Zhao, Rui; Guo, Xing; Wang, ShuoWen; Tu, HaoYang; Yang, Fan	2021	Identification and Therapeutic Outcome Prediction of Cervical Spondylotic Myelopathy Based on the Functional Connectivity From Resting-State Functional MRI Data: A Preliminary Machine Learning Study	Retrospective Longitudinal	6	DCM = 53Healthy Controls = 47	fMRI (BOLD)	Functional Connectivity (FC)
53	Yang, Young-Mi; Oh, Jae-Keun; Song, Ji-Sun; Yoo, Woo-Kyoung; Yoo, Je Hyun; Kwak, Yoon Hae; Kim, Seok Woo	2017	The functional relevance of diffusion tensor imaging in comparison to conventional MRI in patients with cervical compressive myelopathy	Prospective Cross-sectional	N/A	DCM = 20	DTI	FA, ADC
54	Zhang, Meng-Ze; Liu, Jian-Fang; Jin, Dan; Wang, Chun-Jie; Zhao, Qiang; Lang, Ning; Yuan, Hui-Shu; Ou-Yang, Han-Qiang; Liu, Xiao-Guang; Liu, Zhong-Jun; Jiang, Liang; Zhang, Xian-Chang	2021	Utility of Advanced DWI in the Detection of Spinal Cord Microstructural Alterations and Assessment of Neurologic Function in Cervical Spondylotic Myelopathy Patients	Retrospective Longitudinal	3	DCM = 48Healthy Controls = 36	DTI	FA
55	Xiangshui, M.; Xiangjun, C.; Xiaoming, Z.; Qingshi, Z.; Yi, C.; Chuanqiang, Q.; Xiangxing, M.; Chuanfu, L.; Jinwen, H.	2010	3 T magnetic resonance diffusion tensor imaging and fibre tracking in cervical myelopathy	Prospective Cross-sectional	N/A	DCM = 84Healthy Controls = 21	DTI	FA, ADC
56	He, Zhen; Wang, Nan; Kang, Liqing; Cui, Jiaolong; Wan, Yeda	2020	Analysis of pathological parameters of cervical spondylotic myelopathy using magnetic resonance imaging	Prospective Cross-sectional	N/A	DCM = 31Healthy Controls = 8	DTI	FA
57	Mamata, Hatsuho; Jolesz, Ferenc A.; Maier, Stephan E.	2005	Apparent diffusion coefficient and fractional anisotropy in spinal cord: age and cervical spondylosis-related changes	Prospective Cross-sectional	N/A	DCM = 79Healthy Controls = 11	DTI	FA, ADC
58	Zheng, Weipeng; Chen, Haoyi; Wang, Ning; Jiang, Xin; Liang, YingJie; Xiao, Wende; Zhong, Bofu; Ju, Hongbin; Luo, Junnan; Wen, Shifeng; Xiong, Weifeng	2018	Application of Diffusion Tensor Imaging Cutoff Value to Evaluate the Severity and Postoperative Neurologic Recovery of Cervical Spondylotic Myelopathy	Retrospective Longitudinal	12 to 24	DCM = 61	DTI	ADC, MD
59	Kanchiku, T.; Imajo, Y.; Suzuki, H.; Yoshida, Y.; Nishida, N.; Taguchi, T.; Suetomi, Y.; Nishijima, S.	2016	Application of diffusion tensor imaging for the diagnosis of segmental level of dysfunction in cervical spondylotic myelopathy	Retrospective Cross-sectional	N/A	DCM = 10Healthy Controls = 11	DTI	FA, ADC
60	Uda, Takehiro; Takami, Toshihiro; Tsuyuguchi, Naohiro; Sakamoto, Shinichi; Yamagata, Toru; Ikeda, Hidetoshi; Nagata, Takashi; Ohata, Kenji	2013	Assessment of cervical spondylotic myelopathy using diffusion tensor magnetic resonance imaging parameter at 3.0 tesla	Prospective Cross-sectional	N/A	DCM = 26Healthy Controls = 30	DTI	FA, MD
61	Rajasekaran, S.; Kanna, Rishi M.; Balamurali, Gopalakrishnan; Shetty, Ajoy Prasad; Yerramshetty, Janardhan S.; Chittode, Vishnuprasath S.	2014	The assessment of neuronal status in normal and cervical spondylotic myelopathy using diffusion tensor imaging	Prospective Cross-sectional	N/A	DCM = 35Healthy Controls = 40	DTI	ADC
62	Maier, Ilko L; Hofer, Sabine; Eggert, Eva; Schregel, Katharina; Psychogios, Marios-Nikos; Frahm, Jens; Bähr, Mathias; Liman, Jan	2020	T1 Mapping Quantifies Spinal Cord Compression in Patients With Various Degrees of Cervical Spinal Canal Stenosis	Prospective Cross-sectional	N/A	DCM = 31Healthy Controls = 10	Quantitative T1	T1
63	Albistegui-Dubois, Richard; Marehbian, Jonathan; Newton, Jennifer M.; Dong, Yun; Holly, Langston T.; Yan, Xiaohong; Dobkin, Bruce H.	2008	Compensatory cerebral adaptations before and evolving changes after surgical decompression in cervical spondylotic myelopathy: Laboratory investigation	Prospective Longitudinal	6	DCM = 8Healthy Controls = 6	fMRI (BOLD)	Volume of Activation (VOA)
64	Hori, Masaaki; Fukunaga, Issei; Masutani, Yoshitaka; Nakanishi, Atsushi; Shimoji, Keigo; Kamagata, Koji; Asahi, Koichi; Hamasaki, Nozomi; Suzuki, Yuriko; Aoki, Shigeki	2012	New diffusion metrics for spondylotic myelopathy at an early clinical stage	Prospective Cross-sectional	N/A	DCM = 50	DTI	FA, ADC
65	Vedantam, Aditya; Rao, Avinash; Kurpad, Shekar N.; Jirjis, Michael B.; Eckardt, Gerald; Schmit, Brian D.; Wang, Marjorie C.	2017	Diffusion Tensor Imaging Correlates with Short-Term Myelopathy Outcome in Patients with Cervical Spondylotic Myelopathy	Prospective Longitudinal	3	DCM = 27	DTI	FA
66	Wang, Kun; Chen, Zhi; Shen, Hongxing; Zhang, Fan; Song, Qingxin; Hou, Canglong; Tang, Yixing; Wang, Jun; Chen, Shiyue; Bian, Yun; Hao, Qiang	2017	Evaluation of DTI Parameter Ratios and Diffusion Tensor Tractography Grading in the Diagnosis and Prognosis Prediction of Cervical Spondylotic Myelopathy	Prospective Longitudinal	12	DCM = 93Healthy Controls = 36	DTI	FA, ADC
67	Sato, T.; Horikoshi, T.; Watanabe, A.; Uchida, M.; Ishigame, K.; Araki, T.; Kinouchi, H.	2012	Evaluation of cervical myelopathy using apparent diffusion coefficient measured by diffusion-weighted imaging	Prospective Longitudinal	6	DCM = 66	DTI	ADC
68	Takenaka, Shota; Kan, Shigeyuki; Seymour, Ben; Makino, Takahiro; Sakai, Yusuke; Kushioka, Junichi; Tanaka, Hisashi; Watanabe, Yoshiyuki; Shibata, Masahiko; Yoshikawa, Hideki; Kaito, Takashi	2019	Towards prognostic functional brain biomarkers for cervical myelopathy: A resting-state fMRI study	Prospective Longitudinal	6	DCM = 28Healthy Controls = 28	fMRI (BOLD)	Functional Connectivity (FC)
